# Genome-Scale Mining of Novel Anchor Proteins of *Corynebacterium glutamicum*

**DOI:** 10.3389/fmicb.2021.677702

**Published:** 2022-02-04

**Authors:** Kerui Lin, Nannan Zhao, Youhua Cai, Ying Lin, Shuangyan Han, Suiping Zheng

**Affiliations:** ^1^Guangdong Key Laboratory of Fermentation and Enzyme Engineering, School of Biology and Biological Engineering, South China University of Technology, Guangzhou, China; ^2^Guangdong Research Center of Industrial Enzyme and Green Manufacturing Technology, School of Biology and Biological Engineering, South China University of Technology, Guangzhou, China; ^3^Star Lake Bioscience Co. Inc., Zhaoqing Guangdong, Zhaoqing, China

**Keywords:** *Corynebacterium glutamicum*, genome-scale mining, surface display, anchor protein, gram-positive bacteria

## Abstract

The display of recombinant proteins on the surfaces of bacteria is a research topic with many possible biotechnology applications—among which, the choice of host cell and anchoring motif is the key for efficient display. *Corynebacterium glutamicum* is a promising host for surface display due to its natural advantages, while single screening methods and fewer anchor proteins restrict its application. In this study, the subcellular localization (SCL) predictor LocateP and tied-mixture hidden Markov models were used to analyze all five known endogenous anchor proteins of *C. glutamicum* and test the accuracy of the predictions. Using these two tools, the SCLs of all proteins encoded by the genome of *C. glutamicum 13032* were predicted, and 14 potential anchor proteins were screened. Compared with the positive controls NCgl1221 and NCgl1337, three anchoring proteins—NCgl1307, NCgl2775, and NCgl0717—performed better. This study also discussed the applicability of the anchor protein screening method used in this experiment to other bacteria.

## Background

Bacteria that display recombinant proteins on their surfaces play an important role in many biotechnology applications. The choice of host cells and anchoring motifs is essential for the effective display of passenger proteins on cell surfaces. As a host for surface display, *Corynebacterium glutamicum* possesses many useful characteristics, and some successful platforms have been reported. Firstly, *C. glutamicum* exhibits low extracellular protease activity, which is conducive to protein display on its surface (Liu et al., 2013, 2016). Secondly, it is robust and has a wide spectrum of natural carbon source substrates, including pentose, hexose, monosaccharides, and toxic aromatic compounds (Qi et al., 2007; Sasaki et al., 2009; Choi et al., 2019). In addition, *C. glutamicum* has strong tolerance to a variety of inhibitors and weak carbon catabolite repression. Surface display can endow engineered *C. glutamicum* with new functions, expanding their substrate spectra and strengthening the impact of degradation and utilization on renewable biomass (Choi et al., 2019; Zhang et al., 2020).

In order to display proteins on the surface of *C. glutamicum*, several anchoring motifs have been used. At present, the display system of *C. glutamicum* only has three types of anchor proteins: foreign protein (PgsA), mycoloylated proteins [NCgl1337, NCgl0933 (PorB), NCgl0932 (PorC), PorH (NCgl number of PorH have not been assigned)], and membrane protein (NCgl1221, mechanosensitive channel, MscCG) (Tateno et al., 2007b,^2009^; Choi et al., 2018; Inui and Toyoda, 2020). These recombinant strains with anchor proteins have been modified to display carbohydrate-active enzymes (CAZy), such as amylase (Tateno et al., 2007a), glucanase (Ryu and Karim, 2011), glucosidase (Muñoz-Gutiérrez et al., 2012), cellulase complex (Kim et al., 2014) etc., which enable the engineered strains to degrade and utilize cheap biomass. However, similar to most other bacterial hosts, the choice of anchoring motif is critical to the display efficiency of *C. glutamicum*. In order to increase the versatility of the *C. glutamicum* cell surface display technology, it is necessary to develop new and efficient anchoring motifs.

In recent years, the screening of the anchoring proteins of *C. glutamicum* has been based mainly on partial screening rather than direct genome-wide screening of the characteristics of anchor proteins—for example, the process of screening Ncgl1337 as the anchor protein was to identify and analyze the mycolic acid layer protein by SignalP and tied-mixture hidden Markov models (TMHMM) and then display the reporter for further analysis and verification (Choi et al., 2018). The process of screening porin anchor proteins was similar to that (Tateno et al., 2009; Choi et al., 2018). It is worth mentioning that the mining of anchor proteins in other Gram-positive bacteria is generally based on a certain subcellular level or anchoring domain, such as membrane-associated protein (has a transmembrane domain or lipoprotein) or cell wall-associated protein [has a C-terminal Leu-Pro-X-Thr-Gly (LPXTG) motif or cell wall-binding domain] (Desvaux et al., 2006; Fischetti, 2019). These research methods are also used in *Staphylococcus aureus* (Foster, 2019), *Lactococcus lactis* (Plavec et al., 2019), and *Bacillus subtilis* (Guoyan et al., 2019), which are currently the most studied Gram-positive bacteria for anchor proteins.

With recent advancements in bioinformatics technology and the accumulation of a large volume of genome sequencing data as well as the demand for new and efficient anchor proteins, it is necessary to develop new and high-throughput anchor protein mining methods. The optimal properties of anchor proteins should have effective signal peptides or transport signals to allow the fusion protein to pass through the inner membrane. They should have a strong anchoring structure and be compatible with the foreign sequence to be inserted or fused, and they also should resist the attack of the protease present in the periplasmic space or the medium (Lee et al., 2003). In order to identify secreted proteins on the genome scale, numerous subcellular proteomics studies have been carried out (Bumann et al., 2002; Wu et al., 2008; Sharma and Bisht, 2017). In comparison, computational prediction is a relatively fast, auxiliary alternative to microbiological experimental methods that helps to determine SCL and analyze protein function. In recent decades, computational methods and machine learning methods have also become more prominent. Various SCL predictors have been developed, such as SignalP (Almagro Armenteros et al., 2019), PSORTm (Peabody et al., 2020), and LocateP (Zhou et al., 2008). These predictors are designed to identify and classify SCLs based on the Swiss-Prot database or metagenomic sequences. Among them, LocateP can distinguish seven different SCLs within bacteria on the genome scale: intracellular, multi-transmembrane, N-terminally membrane anchored, C-terminally membrane anchored, lipid-anchored, LPXTG-type cell wall-anchored, and secreted/released proteins. Moreover, LocateP can distinguish pathways of general secretion (Sec)- or twin-arginine translocation (Tat)-dependent secretion. LocateP was tested on data sets extracted from the literature, and its accuracy has always been greater than 90%. In addition, researchers have used LocateP to predict the SCLs of all proteins encoded by the genomes of Gram-positive bacteria (Zhou et al., 2008). On the other hand, TMHMM, based on a hidden Markov model approach, can predict the full topology of a protein and visualize its transmembrane structure (Krogh et al., 2001). The application of these tools can greatly facilitate the prediction and mining of anchor proteins.

In this study, we reported a novel method for screening anchor proteins of *C. glutamicum* ATCC13032 based on genome-scale mining. This method attempts to use the SCL prediction tools LocateP and TMHMM to screen the anchor proteins in the genome of *C. glutamicum*. First, we analyzed the five known endogenous anchor proteins of *C. glutamicum* to test the accuracy of prediction of the software packages. Based on the results obtained, the anchor proteins on the genome of *C. glutamicum* ATCC13032 were predicted and analyzed. We screened 25 possible anchor proteins and used enhanced green fluorescent protein (EGFP) and mCherry as reporters to verify the potential anchor proteins and then compared the performance of these two reporters. Then, through the display of α-amylase, the practicality of displaying three highly effective anchor proteins on the surface of *C. glutamicum* was examined. Finally, we discussed the general applicability of this screening method for use with Gram-positive bacteria.

## Materials and Methods

### Reagents, Primers, and Growth Conditions

The reagents and primers used in this study are listed in Supplementary Tables 1, 2. *Escherichia coli* Top10 was used as cloning host for plasmid construction, and *C. glutamicum* ATCC 13032 was used for gene editing. *E. coli* was cultured in LB medium (10 g L^–1^ peptone, 5 g L^–1^ yeast extract, and 10 g L^–1^ NaCl) at 37°C, and *C. glutamicum* ATCC 13032 was cultured in BHISG medium (37 g L^–1^ brain heart infusion, 9.1 g L^–1^ sorbitol, and 10 g L^–1^ glucose) at 30°C. For resistant strains, antibiotics were added as follows: final concentration of 50 μg L^–1^ kanamycin for *E. coli* and 25 μg L^–1^ kanamycin for *C. glutamicum*. In addition, the concentration of isopropyl-βd-thiogalactoside (IPTG) used to induce the expression of α-amylase was 0.5 mM.

### Application of Bioinformatics Technology in the Screening of Anchor Proteins

As mentioned earlier, several endogenous anchor proteins of *C. glutamicum* have been described, namely, NCgl1337, NCgl1221, PorB, PorC, and PorH. In this study, we downloaded the protein sequences of these known endogenous anchor proteins of *C. glutamicum* in FASTA format from the National Center for Biotechnology Information website^1^ and subjected them to LocateP and TMHMM for SCL prediction analysis and transmembrane structure prediction (Table 1, Supplementary Figure 1, and Supplementary Table 3). Next, we analyzed and studied whether the predicted results met the screening requirements of anchor proteins and then used these two tools to predict and screen potential anchor proteins in the genome of *C. glutamicum*.

**TABLE 1:** The prediction results of the known endogenous anchor proteins of *C. glutamicum* made by LocateP: (1) Intracellular probability score: range −1 to +1, threshold: 0.5.

Anchor protein	Protein ID	Product	Pathway	Cellular destination	Subcellular localization	Intracellular possibility	Signal peptide possibility	N-anchored possibility	Cleavage site
NCgl1337	WP_00385 8702.1	SGNH/GDSL hydrolase family protein	Sec-(SPI)	Extracellular	@Secretory (released) (with CS)	0.17	1	−2	PATAQSSG
NCgl1221	WP_01101 4245.1	Mechano sensitive ion channel	Sec-(SPI)	Membrane	@Multi-transmembrane	0.17	−1	1	No cleavage site
NCgl0933	WP_00385 6752.1	PorB	Sec-(SPI)	Extracellular	@Secretory (released) (with CS)	−0.17	1	−1	FAAPASAS
NCgl0932	WP_00385 6749.1	PorC	Sec-(SPI)	Extracellular	@Secretory (released) (with CS)	−0.17	1	−1	PSASAQDF
PorH	WP_01126 5995.1	PorH	No pathway	Cytoplasmic	@Intracellular	1	−1	−1	No cleavage Site

*Values higher than 0.5 increase the probability of the protein occurring in the cell; (2) Extracellular probability score: range −1 to +1, threshold: 0.5. The higher the score, the more likely the protein will be transported to the non-cytoplasmic compartment; (3) N-anchor probability score: no range, threshold: 0. The higher the score, the higher the probability that the protein is retained on the membrane by its N-terminus. A negative score indicates that the protein is very likely to be secreted or released from the membrane (Zhou et al., 2008).*

In the next experiment, we downloaded the protein sequence of the *C. glutamicum* ATCC13032 (GCF_000011325.1, from NCBI bioproject PRJNA307, reference sequence: NC_003450) genome in FASTA format^2^ and then subjected the protein sequence to LocateP and TMHMM for prediction and analysis. Then, based on the characteristics of the anchor proteins, the parameter standards for anchor protein screening were formulated (Table 2), and these two tools were then used to predict and screen potential anchor proteins.

**TABLE 2:** Screening criteria for potential anchor proteins in this experiment.

Subcellular localization	Length	Signal peptide probability (LocateP parameter)	TMHMM parameter	N-anchored probability (LocateP parameter)	Total probability of N-in (TMHMM parameter)
@C-terminally anchored (with CS)	<600	Choose larger parameters	Outside probability of first 20 AA on C-side >0.5; outside probability <0.5		
@Multi-transmembrane (lipid-modified N-termini)	<600		Outside probability of first 20 AA of C-terminal or N-terminal >0.5; outside probability <0.5	Choose larger parameters	
@Lipid-anchored	<600		Outside probability of first 20 AA of C-terminal or N-terminal >0.5; outside probability <0.5	Choose larger parameters	
@Multi-transmembrane	<600		Outside probability of first 20 AA of C-terminal or N-terminal >0.5; outside probability <0.5		
@N-terminally anchored (no CS)	<600		Outside probability of first 20 AA at N-terminal end >0.5; outside probability <0.5	Choose larger parameters	>0.8
@Secretory (released) (with CS)	<600		Outside probability of first 20 AA of C-terminal or N-terminal >0.5; outside probability <0.5	Choose larger parameters	>0.8

*These screening criteria are applicable to anchor proteins anchored at the N-terminus, and the opposite is true for anchor proteins anchored at the C-terminus.*

### Construction of the Surface Display System of Recombinant *C. glutamicum*

The amplification and identification of gene fragments were performed by KOD DNA polymerase (TOYOBO, Japan) and Taq DNA polymerase (Thermo Fisher Scientific, Waltham, MA, United States). The Seamless Assembly Cloning Kit (Clone Smarter, United States) was used to assemble the plasmids using the principle of homologous recombination. In all expression vectors, the ribosome binding site (AAAGGAGGCCCTTCAG) is added before the open reading frame of the fusion protein. The constructed plasmids were verified by DNA sequencing (Shanghai Generay, Shanghai, China). All primers were synthesized by Shanghai Generay Biotech Co., Ltd. (Shanghai, China).

The construction of the *C. glutamicum* surface display system used pEC-XK99e as the carrier and EGFP, mCherry, and α-amylase as the reporters, respectively. The gene (*amyE*) of α-amylase (BSU_03040) was derived from *B. subtilis* 168. The N-terminal fusion or C-terminal fusion was determined according to the characteristics of the anchor proteins screened. Among these 25 anchor proteins, NCgl0550, NCgl0633, and NCgl2291 are C-terminal fusions, and the rest are N-terminal fusions (the term C-terminal or N-terminal fusion here refers to the way the anchor protein anchors on the cell surface, not the way the anchor protein anchors the passenger protein). The N-terminal/C-terminal of the anchor protein has a FLAG (DYKDDDDK, where D = aspartic acid, Y = tyrosine, and K = lysine) tag.

**FIGURE 1:**
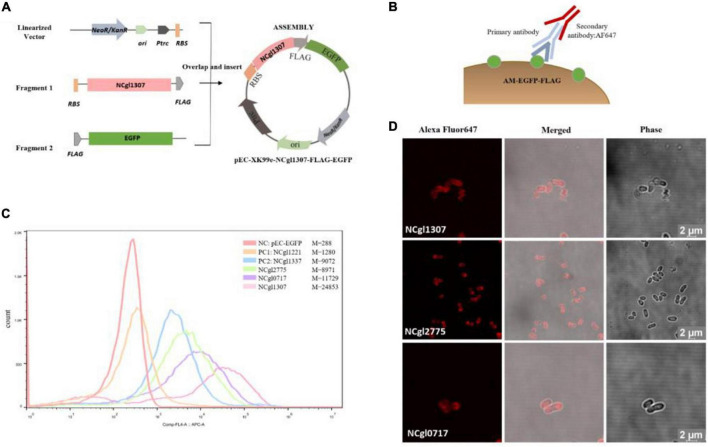
Screen and confirmation of anchoring protein display on the cell surface by displaying EGFP. **(A)** Construction of recombinant *Corynebacterium glutamicum* surface display plasmid with EGFP as passenger protein. The construction of all the expression vectors of passenger protein EGFP was constructed using the same cloning strategy with a different PCR primer set. Take the construction of Ncgl1307-EGFP as an example. Using the genomic DNA of *C. glutamicum* ATCC13032 as a template and using 5am F and 5am R as primers, the Ncgl1307 gene sequence fragment 1 was amplified by PCR; using the EGFP gene as a template and using EGFP F-N site and EGFP R-N site as primers, the target protein EGFP gene fragment 2 was amplified; using pEC-XK99e as a template and pEC F and pEC R as primers, the gene sequence linearized vector of pEC-XK99e was amplified. Finally, this three fragments were assembled into a plasmid by Seamless Assembly Cloning Kit. **(B)** Schematic diagram of the immunofluorescence reaction. Anti-flag monoclonal antibody and Alexa Fluor 647-labeled antibody were added to the EGFP-FLAG. **(C)** Flow cytometry analysis. The peak figures of different colors from left to right represent the fluorescent signal intensities of cells harboring pEC-EGFP, Ncgl1221-EGFP, Ncgl1337-EGFP, NCgl2775-EGFP, NCgl0717-EGFP, or NCgl1307-EGFP, respectively. Of these, cells harboring pEC-EGFP were the negative control (NC_–EGFP_), and cells harboring Ncgl1221-EGFP and Ncgl1337-EGFP were the positive control 1 (PC1_–EGFP_) and the positive control 2 (PC2_–EGFP_), respectively. M is mean value of Comp-FL-A:APC-A_Area. **(D)** Image observed on a confocal microscope based on immunofluorescence reaction. The red fluorescence comes from the fluorescence of the Alexa Fluor 647-labeled antibody on the flag label (667 nm—excitation, 651 nm—emission).

**FIGURE 2:**
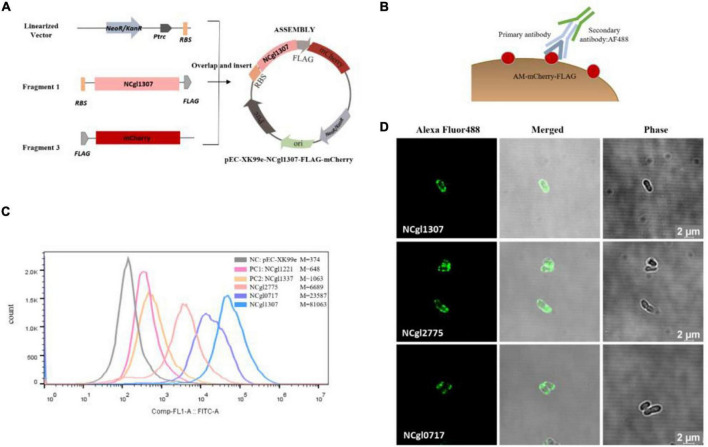
Screening and confirmation of anchoring protein display on the cell surface by displaying mCherry. **(A)** Construction of recombinant *Corynebacterium glutamicum* surface display system with mCherry as passenger protein. Take the construction of Ncgl1307-mCherry as an example. Using mCherry gene as template and mCherry-pEC F and mCherry-pEC R as primers, mCherry gene fragment 3 was amplified. Then, Seamless Assembly Cloning Kit was used to assemble fragment 1, fragment 3, and linearized vector (Figure 1A). **(B)** Schematic diagram of the immunofluorescence reaction. Anti-flag monoclonal antibody and Alexa Fluor 488-labeled antibody were added to the mCherry-FLAG. **(C)** Flow cytometry analysis. The peak figures of different colors from left to right represent the fluorescent signal intensities of cells harboring pEC-XK99e, Ncgl1221-mCherry, Ncgl1337-mCherry, NCgl2775-mCherry, NCgl0717-mCherry, or NCgl1307-mCherry, respectively. Of these, cells harboring pEC-XK99e were the negative control (NC_–mCherry_), and cells harboring Ncgl1221-mCherry and Ncgl1337-mCherry were the positive control 1 (PC1_–mCherry_) and the positive control 2 (PC2_–mCherry_), respectively. *M* is the mean value of Comp-FL-A:FITC-A_Area. **(D)** Image observed on a confocal microscope. The green fluorescence comes from the fluorescence of the Alexa Fluor 488-labeled antibody on the flag label (488 nm—excitation, 520 nm—emission).

**FIGURE 3:**
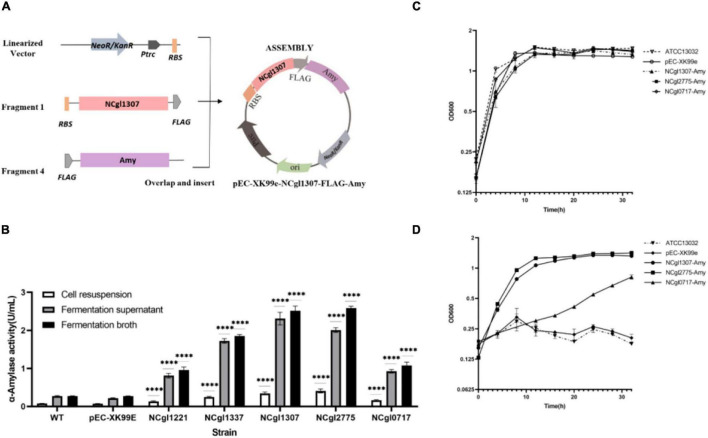
Display of α-amylase on the cell surface using NCgl1307, NCgl2775, or NCgl0717 as anchoring motifs. **(A)** Construction and identification of a recombinant *Corynebacterium glutamicum* surface display system with α-amylase as passenger protein. Take the construction of Ncgl1307-amylase as an example. Using the amylase gene as a template and Amy F and Amy R as primers, the target protein amylase gene fragment 4 was amplified. Then, Seamless Assembly Cloning Kit was used to assemble fragment 1, fragment 4, and linearized vector (Figure 1A). **(B)** α-Amylase activity assay with WT, NC (cells harboring pEC-XK99e), cells harboring NCgl1307-Amy, NCgl2775-Amy, and NCgl0717-Amy, respectively. The data represent the mean value and standard deviation of biological triplicates. Statistical significance, ^*⁣*⁣**^P < 0.0001. **(C)** Growth curve of *C. glutamicum* with glucose as the sole carbon source, including WT, pEC-XK99e, NCgl1307-Amy, NCgl2775-Amy, and NCgl0717-Amy (representing cells harboring pEC-XK99e, NCgl1307-Amy, NCgl2775-Amy, or NCgl0717-Amy, respectively). **(D)** Growth curve of *C. glutamicum* with starch as the sole carbon source, including WT, pEC-XK99e, NCgl1307-Amy, NCgl2775-Amy, and NCgl0717-Amy (representing cells harboring pEC-XK99e, NCgl1307-Amy, NCgl2775-Amy, or NCgl0717-Amy, respectively). The data represent the mean value and standard deviation of biological triplicates.

The construction of all the expression vectors was constructed using the same cloning strategy with a different PCR primer set. For the expression vector of passenger protein EGFP, here is the construction of Ncgl1307-EGFP as an example (Figure 1A). Using the genomic DNA of *C. glutamicum* ATCC13032 as a template and using 5am F and 5am R as primers, the Ncgl1307 gene sequence fragment 1 was amplified by PCR; using the EGFP gene as the template and using EGFP F-N site and EGFP R-N site as primers, the target protein EGFP gene fragment 2 was amplified; using pEC-XK99e as a template and pEC F and pEC R as primers, the gene sequence linearized vector of pEC-XK99e was amplified. Finally, these three fragments were assembled into a plasmid by Seamless Assembly Cloning Kit. For the expression vector where the passenger protein is mCherry, here is the construction of Ncgl1307-mCherry as an example (Figure 2A). Using mCherry gene as template and mCherry-pEC F and mCherry-pEC R as primers, mCherry gene fragment 3 was amplified. Then, Seamless Assembly Cloning Kit was used to assemble fragment 1, fragment 3, and linearized vector. For the expression vector where the passenger protein is amylase, here is the construction of Ncgl1307-amylase as an example (Figure 3A). Using the amylase gene as a template and Amy F and Amy R as primers, the target protein amylase gene fragment 4 was amplified. Then, Seamless Assembly Cloning Kit was used to assemble fragment 1, fragment 4, and linearized vector.

### Flow Cytometry and Laser Confocal Microscopy of the Recombinant *C. glutamicum* Surface Display System

The recombinant *C. glutamicum* cells were induced and cultured in BHISG medium supplemented with 25 μg/ml kanamycin and 0.5 mM IPTG (final concentration) for 24 h, centrifuged at 6,000 rpm for 10 min, and then resuspended in 1 ml phosphate-buffered saline (PBS), and the optical density at 600 nm (OD_600__nm_) was adjusted to 1. The cells were centrifuged at 6,000 rpm for 10 min and resuspended in 0.2 ml PBS containing 1% bovine serum albumin (BSA). Then, 1 μl of anti-FLAG monoclonal antibodies (1 μg/ml; Sigma, United States) was added, and the mixture was incubated at room temperature for 2 h, with gentle shaking to avoid precipitation. Subsequently, the cells were washed twice with PBS and resuspended with 0.2 ml PBS containing 1% BSA. For the strain whose reporter was EGFP, 1 μl of 2 μg/μl Alexa Fluor 647-labeled goat anti-mouse IgG antibody [AF647, Alexa Fluor 647-labeled Goat Anti-Mouse IgG (H + L), Beyotime, China] was added, incubated at room temperature for 1 h, and then shaken continuously to suspend it. Finally, a BD Accuri™ C6 Plus Flow Cytometer (BD Biosciences, San Jose, CA, United States) was used to measure the fluorescence intensity. A total of 50,000 cells per sample was collected. Subsequently, the sample was centrifuged and resuspended, then coated on a microscope slide, and finally observed through a confocal microscope (Leica TCS SP8, Wetzlar, Germany). The image was acquired at an excitation wavelength of 488 nm and an emission wavelength of 520 nm. For strains whose reporter was mCherry, an Alexa Fluor 488-labeled goat anti-mouse IgG antibody [AF 488, Alexa Fluor 488-labeled Goat Anti-Mouse IgG (H + L), Beyotime, China] was used. A confocal microscope was used at the excitation wavelength of 667 nm and the emission wavelength of 651 nm to acquire the images. For flow cytometry analysis, Comp-FL1-A:APC-A was used to detect AF647, and Comp-FL1-A:FITC-A was used to detect AF 488. The data of flow cytometry and confocal microscopy were analyzed and processed by GraphPad Prism 8 and LAS X software, respectively.

### Protein Fractionation and Analysis

*Corynebacterium glutamicum* harboring NCgl1307-Amy or NCgl2775-Amy or NCgl0717-Amy was selected and induced by 0.5 mM IPTG. The induced cells grew at 30°C with continuous shaking for 24 h before the cells were harvested. The cell surface fraction was prepared as described by Tateno et al. (2007b). The supernatant samples containing only cell wall-associated proteins were analyzed by sodium dodecyl sulfate-polyacrylamide gel electrophoresis (SDS-PAGE) using 8% gel. Then, the proteins were electroblotted onto a nitrocellulose membrane (Immobilon-P Membrane, PVDF, 0.45 mm; EMD Millipore Corporation, Billerica, MA, United States). As the molecular weight marker, prestained protein standards (26616; Thermo Fisher Scientific, United States) were used. The membrane was blocked for 1 h at 37°C with Tris-buffered saline (TBS) with Tween-20 containing 5% BSA. After washing for five times with TBS, the membrane was incubated with the anti-FLAG monoclonal antibody (Sigma, United States) at a dilution of 1:5,000 and the secondary Goat Anti-mouse IgG (H&L) (HRP) antibody (Aksomics, China) at a dilution of 1:5,000. The membrane was then stained with ultra-sensitive ECL chemiluminescence substrate (BL520A, Biosharp, China) according to the protocol of the manufacturer. Finally, ChemiScope 3300 mini integrated chemiluminescence imaging system (Clinx, China) was used for detection.

### Determination of Amylase Activity and Growth Curve

The amylase activity is determined by using the EnzChek^®^ Ultra Amylase Assay Kit (Invitrogen, United States), and the specific steps used relate to the method used by Choi et al. (2018). The reaction mixture was added on a black 96-well plate (100 μl/well) (Corning, United States). Fluorescence was measured by a TECAN Infinite M200 ELISA plate reader (Tecan, Switzerland), using excitation at 485 nm and fluorescence detection at 520 nm. One unit (U) of α-amylase activity was defined as the amount of enzyme required to liberate 1.0 mg of glucose from starch in 3 min, at pH 6.9 and 20°C. The data was analyzed by GraphPad Prism 8 software. Each incubation was conducted in triplicate, and the results are presented as mean ± standard error from at least three independent experiments.

The strains picked from the plate were inoculated into BHISG and cultured overnight at 30°C and 280 rpm. CGXII medium (Supplementary Table 1) was used to determine the growth curve, and glucose (4%; Damao, China) or soluble starch (4%; Damao, China) was used as the only carbon source. The initial OD_600nm_ of cell density was 0.1–0.3, and the strain was cultured at 30°C and 280 rpm in 12-well plates. The OD_600_ optical density was measured every 4 h for 32 h. During the cultivation process, final concentrations of 25 μg/ml kanamycin and 0.5 mM IPTG were added to the resistant strains of *C. glutamicum*. The data was analyzed by GraphPad Prism 8 software. Each incubation was conducted in triplicate, and the results are presented as mean ± standard error from at least three independent experiments.

## Results and Discussion

### Predictions of the Known Endogenous Anchor Proteins

Previously, several anchor proteins of *C. glutamicum* have been described, including NCgl1337, NCgl1221, PorB, PorC, and PorH. All except NCgl1221 are located in the mycolic acid layer. Of these five proteins, PorB, PorC, and PorH are porins, and they function as channels for transporting small molecules in the mycolic acid layer. NCgl1221 is a mechanosensitive channel and the main glutamate exporter of *C. glutamicum*, which is located on the cell membrane (Issa et al., 2017; Nakayama et al., 2019). To test the accuracy of the predictions made by LocateP and TMHMM, we analyzed the five known endogenous anchor proteins mentioned above by these two software packages. The prediction of LocateP showed that these proteins all belong to Sec-(SPI) secretion pathways and locate in the extracellular or cell membrane, except PorH (Table 1). The analysis of TMHMM also indicated that the parameters of these proteins are suitable for anchor proteins, including signal peptides and transmembrane structures (Supplementary Figure 1 and Supplementary Table 3). Therefore, the predictions of the known anchor proteins made by LocateP and TMHMM of *C. glutamicum* are quite reliable.

In order to screen the anchor proteins in *C. glutamicum* ATCC13032, the amino acid (AA) sequence of the predicted proteins of *C. glutamicum* ATCC13032 were subjected in the FASTA format to LocateP and TMHMM. Then, based on the characteristics of these subcellular compartments and the requirements for screening anchor proteins in this experiment, we developed several screening criteria (Table 2). In view of the metabolic burden caused by the excessive molecular weight of the anchoring protein, proteins below 600 AA were selected. As for the signal peptide, those parameters of a larger signal peptide were selected in order to increase the possibility of forming an anchor protein. For N-terminal anchor proteins, to ensure that the reporter or other functional proteins fused to the anchor protein can extend outside the cell, the inside probability parameter of the first 20 AA at the N-terminal end should be >0.5, and the outside probability should be <0.5. For @N-terminally anchored (no CS) and @Secretory (released) (with CS) N-anchored probability (LocateP parameter) and total probability of N-in (TMHMM2.0 parameter), larger parameters should be chosen to increase the probability of N-terminal anchoring. These screening rules are reversed for C-terminally anchor proteins. Based on these screening criteria, we screened 25 potential anchor proteins with high scores from the *C. glutamicum* ATCC13032 genome (Table 3 and Supplementary File 1).

**TABLE 3:** The 25 potential anchor proteins screened in this experiment based on the predicted results of LocateP and tied-mixture hidden Markov models.

	Identifier	Locus tag	Pathway	Cellular destination	Subcellular localization	Intracellular possibility	Signal peptide possibility	N-anchored possibility	Cleavage site	Potential anchored site
1	WP_011013799.1 hypothetical protein	NCgl0550	Sec-(SPI)	Membrane	@C-terminally anchored (with CS)	0	1	−2	PTASAATL	C site
2	WP_011013739.1 type VII secretion-associated serine protease mycosin	NCgl0633	Sec-(SPI)	Membrane	@C-terminally anchored (with CS)	0	1	−2	TRAQEVEA	C site
3	WP_011014951.1 cytochrome c oxidase subunit II	NCgl2115	Sec-(SPII)	Membrane	@Multi-transmembrane (lipid-modified N-termini)	0	1	1	LAMAGCE	N site
4	WP_011265759.1 gi| 62390400| ref| YP_225802.1| hypothetical protein cg1712	NCgl1460	Sec-(SPII)	Extracellular	@Lipid-anchored	0.17	1	1	LLLSACT	N site
5	WP_011014306.1 hypothetical protein	NCgl1307	Sec-(SPII)	Extracellular	@Lipid-anchored	−0.33	1	1	FVLSGCG	N site
6	WP_011014779.1 glutamate ABC transporter substrate-binding protein	NCgl1876	Sec-(SPII)	Extracellular	@Lipid-anchored	−0.33	1	1	VTLTACG	N site
7	WP_011265985.1 twin-arginine translocation signal domain-containing protein	NCgl2562	Possibly Tat/Sec-(SPII)	Extracellular	@Lipid-anchored	−0.33	1	1	ATLAACA	N site
8	WP_011013364.1 sensor histidine kinase	NCgl0067	Sec-(SPI)	Membrane	@Multi-transmembrane	0.17	−1	2	No Cleavage Site	N site
9	WP_004567665.1 MULTISPECIES: DUF4233 domain-containing protein	NCgl2291	Sec-(SPI)	Membrane	@Multi-transmembrane	0.17	−1	1	No cleavage site	C site
10	WP_01101483.1 family transporter	NCgl1147	Sec-(SPI)	Membrane	@Multi-transmembrane	0.17	−1	1	No cleavage site	N site
11	WP_011014865.1 ABC transporter ATP-binding protein	NCgl1998	Sec-(SPI)	Membrane	@Multi-transmembrane	0.17	−0.5	1	No cleavage site	N site
12	WP_003859459.1 MULTISPECIES: HlyC/CorC family transporter	NCgl2206	Sec-(SPI)	Membrane	@Multi-transmembrane	0.17	0	0	No cleavage site	N site
13	WP_003863539.1 MULTISPECIES: potassium channel family protein	NCgl0743	Sec-(SPI)	Membrane	@Multi-transmembrane	0.17	−1	−1	No cleavage site	N site
14	WP_011013342.1 penicillin-binding protein 2	NCgl0042	Sec-(SPI)	Membrane	@N-terminally anchored (no CS)	0.17	1	7	No cleavage site	N site
15	WP_011013540.1 TlpA family protein disulfide reductase	NCgl0289	Sec-(SPI)	Membrane	@N-terminally anchored (no CS)	0.17	1	7	No cleavage site	N site
16	WP_011014270.1 SRPBCC family protein	NCgl1250	Sec-(SPI)	Membrane	@N-terminally anchored (no CS)	0.17	1	7	No cleavage site	N site
17	WP_011015453.1 cutinase family protein	NCgl2775	Sec-(SPI)	Membrane	@N-terminally anchored (no CS)	0.17	1	7	No cleavage site	N site
18	WP_003853779.1 MULTISPECIES: DUF4247 domain-containing protein	NCgl2610	Sec-(SPI)	Membrane	@N-terminally anchored (no CS)	0.17	1	5	No cleavage site	N site
19	WP_011013420.1 hypothetical protein	NCgl0136	Sec-(SPI)	Extracellular	@Secretory (released) (with CS)	0.17	1	−1	IAATPATA	N site
20	WP_011013818.1 CAP domain-containing protein	NCgl0661	Sec-(SPI)	Extracellular	@Secretory (released) (with CS)	0.17	1	−1	PSAHAFTA	N site
21	WP_011013866.1 hypothetical protein	NCgl0717	Sec-(SPI)	Extracellular	@Secretory (released) (with CS)	0.17	1	−1	DIATSTTT	N site
22	WP_011014348.1 copper transporter	NCgl1361	Sec-(SPI)	Extracellular	@Secretory (released) (with CS)	0.17	1	−1	GIAFGTYV	N site
23	WP_011014599.1 LysM peptidoglycan-binding domain-containing protein	NCgl1682	Sec-(SPI)	Extracellular	@Secretory (released) (with CS)	0.17	1	−1	GGSGVTFL	N site
24	WP_042383306.1 hypothetical protein	NCgl2577	Sec-(SPI)	Extracellular	@Secretory (released) (with CS)	0.17	1	−1	VVALRGGS	N site
25	WP_003858490.1 resuscitation-promoting factor	NCgl0872	Sec-(SPI)	Extracellular	@Secretory (released) (with CS)	0.17	1	−2	VTAAATKK	N site

### Construction of the Surface Display System Using EGFP as the Passenger Protein

In the cells of organisms of the three-domain system (archaea, bacteria, and eukaryotes), secretory proteins are usually synthesized in the form of a precursor protein, carrying a split N-terminal signal peptide, which is used to locate the protein in a membrane-embedded export device (Rapoport et al., 1996; Eichler, 2000). *C. glutamicum* has two main protein secretion pathways: Sec-dependent pathway to secrete unfolded proteins and Tat-dependent pathway to secrete folded proteins (Vertès, 2013; Freudl, 2017). Fluorescent proteins are the main cell biology tool for analyzing the subcellular topology of protein, of which EGFP and mCherry are commonly used. EGFP is an excellent reporter of the Tat system in *Streptomyces listeri* (Hamed et al., 2018) and *E. coli* (Lee et al., 2006) because active EGFP can only be secreted after cytoplasmic folding and subsequent translocation through the Tat pathway. Fusion of GFP to a Sec-specific signal peptide resulted in the periplasmic localization of GFP, but in an inactive form (Albiniak et al., 2013). However, the monomeric red fluorescent protein mCherry is functional regardless of the translocation pathway (Speck et al., 2011). In order to eliminate interference by the reporter due to its own secretion pathway and compare the differences between the two commonly used reporters EGFP and mCherry, we used these two reporters to screen the anchor proteins sequentially.

First, we used EGFP as the passenger protein to test the display effect of the 25 potential anchor proteins (Table 3). The potential anchored protein predicted was amplified by PCR and connected to pEC-XK99e through homologous recombination. In order to reduce the impact of space barrier on the screening process, the membrane protein NCgl1221 and the mycolic acid protein NCgl1337 were selected as two positive control of anchor proteins. The strain harboring pEC-EGFP was the negative control.

### Flow Cytometry and Laser Confocal Scanning Microscopy of the Recombinant *C. glutamicum* Surface Display System Based on Immunofluorescence

In the initial experiment, the strain displaying EGFP was resuspended for laser confocal analysis without immunofluorescence reaction. The results showed that most samples could not determine whether EGFP was displayed on the surface due to strong intracellular fluorescence interference (Supplementary Figure 2). Therefore, immunofluorescence reaction was used before fluorescence-activated cell sorting (FACS) and confocal microscopy analysis (Figures 1B, 2B).

The 25 anchor proteins were fused with the EGFP-FLAG, and Ncgl1221 (PC1_–EGFP_) and Ncgl1337 (PC2_–EGFP_) were used as the two positive controls. In order to evaluate the fluorescence localization, we used FACS and confocal fluorescence microscopy based on the immunofluorescence reaction. In FACS analysis, the 14 cells harboring FLAG-tagged fusion-anchored proteins showed a significant drift compared to the negative control (cells harboring pEC-EGFP) (Supplementary Figure 3). In addition, among the 14 recombinant strains, the cells harboring NCgl1307-EGFP, NCgl2775-EGFP, and NCgl0717-EGFP showed a more significant drift than the two positive controls (PC1_–EGFP_ and PC2_–EGFP_) (Figure 1C).

In order to further confirm the localization of fluorescence, we observed these 25 recombinant strains with immunofluorescence under a confocal microscope. The results showed that these 14 recombinant strains mentioned above all showed the red fluorescent signal of the secondary antibody under a laser confocal microscope, including cells harboring NCgl1307, NCgl2775, and NCgl0717 (Figure 1D), which performed well in flow cytometry. The results showed that these 14 proteins could be used as anchor proteins to display EGFP on the surface of *C. glutamicum*.

### Using the Mined Anchor Protein to Display mCherry

The active fluorescent EGFP needs to be secreted through the Tat pathway, while the monomeric red fluorescent protein mCherry is functional regardless of the translocation pathway (Speck et al., 2011). Almost all the 25 potential anchor proteins screened above are secreted through the Sec pathway (Table 3). In order to eliminate the interference from its own secretory pathway when EGFP acts as a reporter, we selected several anchor proteins to display mCherry.

In this experiment, NCgl1307, NCgl2775, NCgl0717, NCgl0743, NCgl1361, and NCgl2610 were fused with mCherry-FLAG. Ncgl1221 (PC1_–mCherry_) and Ncgl1337 (PC2_–mCherry_) were used as the two positive controls of anchor protein. In order to evaluate the fluorescence localization, we used FACS and confocal fluorescence microscopy. In FACS analysis, the protein-anchored cells showed a more significant drift than the negative control (NC, cells harboring pEC-XK99e) (Supplementary Figure 4). In addition, the cells harboring NCgl2775-mCherry, NCgl0717-mCherry, and NCgl1307-mCherry showed a higher fluorescence intensity than the positive controls (cells harboring Ncgl1221-mCherry or Ncgl1337-mCherry) (Figure 2C); these results were similar to those of EGFP. At the same time, when comparing the strain with the reporter as EGFP (Figure 1C), the peak pattern of the recombinant strain harboring NCgl0717-mCherry and NCgl1307-mCherry shifted more obviously (Figure 2D).

### Expression of Anchor Protein—Amy Fusion Protein—on the Cell Surface

In order to explore the potential practical applications of the mined anchor proteins, we used α-amylase (AmyE) of *B. subtilis* 168 as the passenger protein. The expression of the anchor protein—Amy fusion proteins—in *C. glutamicum* was analyzed by SDS-PAGE and Western blot analysis of the cell wall fraction. The molecular size of the individual NCgl1307, NCgl2775, NCgl0717, and Amy proteins is approximately 38, 40 (Meniche et al., 2009), 27, and 73 kDa, respectively. The molecular size of the fusion proteins NCgl1307-Amy, NCgl2775-Amy, and NCgl0717-Amy was therefore predicted to be approximately 111, 113, and 100 kDa, respectively. Bands at the predicted molecular size were observed on Western blot, indicating the successful expression of the fusion proteins in the cell wall fraction (Supplementary Figure 5). However, the sample of the fusion proteins used on Western blot was easily degraded.

Next, the amylase activity and cell growth of display platforms with NCgl1307-Amy, NCgl2775-Amy, and NCgl0717-Amy were analyzed (Figure 3). Compared with the positive controls NCgl1221 and NCgl1337, the enzyme activity of the strain containing NCgl1307 and NCgl2775 was higher than that of the two positive control strains. The enzyme activity of the strain containing NCgl0717 is higher than that of the positive control strain containing NCgl1221 and lower than that of the positive control strain containing NCgl 1337 (Figure 3B). Their enzyme activities of cell resuspension reached 0.34 (52), 0.41 (62), and 0.16 U/ml (25 U/g dry cells), respectively.

In addition, WT and strains harboring pEC-XK99e, NCgl1307-Amy, NCgl2775-Amy, and NCgl0717-Amy have similar growth curves in the glucose medium. Of these, WT has the highest OD_600_ value in the stable phase (Figure 3C). This may be caused by the heavy metabolic burden of recombinant strains affected by plasmids and antibiotics. As negative controls, WT and cells harboring pEC-XK99e did not grow well in the starch medium because of their inability to utilize starch (Figure 3D). In contrast, strains harboring NCgl1307-Amy, NCgl2775-Amy, and NCgl0717-Amy grew much better in the starch medium. Of these, the growth trends of the strains with NCgl1307-Amy and NCgl2775-Amy were similar in both the starch and the glucose media, including the time point for entering the stable phase and the maximum OD_600_ value, which suggested that the strains harboring NCgl1307-Amy and NCgl2775-Amy are well able to utilize starch.

However, the protein sequence of the α-amylase gene (*amyE*, BSU_03040) used in this experiment includes signal peptide (1--27 AA) and propeptide (28--41 AA)^3^. The signal peptide and propeptide sequences will be cleaved off after guiding the transport of the amylase protein across the membrane. This should be the main reason why most amylases are present in the supernatant in this experiment. In subsequent experiments, we plan to remove the gene sequences of the signal peptide and propeptide and further explore the application of the 14 anchored proteins to the display of this α-amylase or other functional proteins.

Moreover, using the protein basic local alignment search tool (BLASTP) and protein functional region analysis, it was found that, among the three anchor proteins screened, NCgl2775 is a membrane protein and possesses comparable esterase and thioesterase activities *in vitro* (Meniche et al., 2009), which means that these enzyme activities may work synergistically with the displayed enzymes. Therefore, the characteristic of this anchor protein may have a more useful application value in the future. NCgl1307 and NCgl0717 are hypothetical proteins, and their functions need to be studied further. The results in BLASTP also showed that these three anchor proteins are also present in many other *C. glutamicum* species, and it is expected that they may have more extensive uses in the future.

## Conclusion and Prospects

In this study, we used the subcellular localization and parameter prediction of LocateP and the transmembrane structure prediction and visualization of TMHMM to screen the anchor proteins in the protein sequence of the *C. glutamicum* genome. A total of 14 potential anchor proteins were screened. Among them, three had a higher rivet efficiency than the previously mined NCgl1221 and NCgl1337 and showed enzyme activities with amylase. These results confirmed the effectiveness of the screening method. At the same time, any protein that can rivet the protein of interest to the cell surface could be used as a potential anchor protein. Therefore, our screening method is likely to be applicable to other Gram-positive bacteria. Moreover, the operations during the screening by LocateP and TMHMM can be carried out according to specific parameters. LocateP has completed the prediction of SCLs of all of the proteins encoded by the Gram-positive bacterial genome (Zhou et al., 2008). Those laid a good foundation for further high-throughput screening of anchor proteins. With advancements in information technology, it is expected that researchers will be able to combine these two tools to develop open-source software specifically for screening the anchor proteins of Gram-positive bacteria. This could help develop more efficient anchor proteins for basic theoretical research and practical industrial applications.

On the other hand, in the screening process of anchor proteins, the reporters EGFP and mCherry have their own characteristics. The different preferences in secretion pathway of these two reporters may have few effects on secretion and anchoring. Meanwhile, the secretion pathway of the signal peptide may play a major role in the secretion and transport of the anchor protein–reporter. It is worth mentioning that, no matter which fluorescent protein is selected, the ideal anchoring effect cannot be directly observed under the confocal laser microscope without immunofluorescence reactions because of the interference from intracellular fluorescence. In summary, functional proteins with FLAG tags against secondary antibodies AF488 might be a good choice in the screening process for anchor proteins. This combination can achieve better results in both FACS and confocal microscopy and directly verify the practicality of anchor proteins.

## Data Availability Statement

The original contributions presented in the study are included in the article/Supplementary Material, further inquiries can be directed to the corresponding author/s.

## Author Contributions

KL and SZ designed and carried out the main studies. SH, NZ, YC, and YL have made a lot of contributions in the experiment design, experiment execution, and manuscript revision. All authors read and approved the final manuscript.

## Conflict of Interest

YC was employed by Star Lake Bioscience Co. Inc. The remaining authors declare that the research was conducted in the absence of any commercial or financial relationships that could be construed as a potential conflict of interest.

## Publisher’s Note

All claims expressed in this article are solely those of the authors and do not necessarily represent those of their affiliated organizations, or those of the publisher, the editors and the reviewers. Any product that may be evaluated in this article, or claim that may be made by its manufacturer, is not guaranteed or endorsed by the publisher.

## Footnotes

1
https://www.ncbi.nlm.nih.gov

2
https://ftp.ncbi.nlm.nih.gov/genomes/refseq/bacteria/Corynebacterium_glutamicum/latest_assembly_versions

3
https://www.uniprot.org/uniprot/P00691

## Supplementary Material

Supplementary Figure 1 Tied-mixture hidden Markov models predicted the results for the transmembrane structure of the known endogenous anchor proteins of *Corynebacterium glutamicum*. **(A)** NCgl1337, **(B)** NCgl1221, **(C)** PorB, **(D)** PorC, and **(E)** PorH.

Supplementary Figure 2 Laser confocal scanning microscopy analysis of the recombinant *Corynebacterium glutamicum* surface display system without immunofluorescence reaction.

Supplementary Figure 3 Flow cytometric analysis of recombinant *Corynebacterium glutamicum* with surface-displayed EGFP based on immunofluorescence reaction (single-comparison chart). The peak image without color filling on the left is the peak image of the negative control strain (NC, cells harboring pEC-EGFP, *M* = 288). The NCgl number indicates the corresponding strain displaying NCgl protein-EGFP. Cells harboring Ncgl1221-EGFP and Ncgl1337-EGFP were the positive control 1 (PC1_–EGFP_) and the positive control 2 (PC2_–EGFP_), respectively. *M* is the mean value of Comp-FL-A:APC-A _Area.

Supplementary Figure 4 Flow cytometry analysis of recombinant *Corynebacterium glutamicum* with surface-displayed mCherry based on immunofluorescence reaction (single-comparison chart). The peak image without color filling on the left is the peak image of the negative control strain (NC, cells harboring pEC-XK99e, *M* = 374). The NCgl number indicates the corresponding strain displaying NCgl protein-mCherry. Cells harboring Ncgl1221-mCherry and Ncgl1337-mCherry were the positive control 1 (PC1_–mCherry_) and the positive control 2 (PC2_–mCherry_), respectively. *M* is the mean value of Comp-FL-A:FITC-A_Area.

Supplementary Figure 5 Western blot analysis of protein production in *Corynebacterium glutamicum*. **(A)** Lanes 8 and 9 represent the cell wall fraction protein of cells harboring NCgl0717-Amy and NCgl1307-Amy, respectively. **(B)** Lane 3 represents the cell wall fraction protein of cells harboring NCgl2775-Amy.

Supplementary Table 1 Reagent used in this study.

Supplementary Table 2 Primers used in this study.

Supplementary Table 3 The prediction results of the known endogenous anchor proteins of *C. glutamicum* made by TMHMM.

Supplementary File 1 Tied-mixture hidden Markov models predicted the results for the 25 possible anchor proteins of *Corynebacterium glutamicum*.
