# Supplementary material for: Genome-Scale Mining of Novel Anchor Proteins of Corynebacterium glutamicum
Source: Front Microbiol. 2022 Feb 4;12:677702. doi: 10.3389/fmicb.2021.677702 (PMC8854784; doi:10.3389/fmicb.2021.677702)
Supplement: Supplementary Figure 1 — Tied-mixture hidden Markov models predicted the results for the transmembrane structure of the known endogenous anchor proteins of Corynebacterium glutamicum. (A) NCgl1337, (B) NCgl1221, (C) PorB, (D) PorC, and (E) PorH. [file Data_Sheet_1.docx]

TMHMM2.0 predictions of 25 possible anchor proteins and their genetic information

1

# WP_011013739.1 Length: 414

# WP_011013739.1 Number of predicted TMHs: 1

# WP_011013739.1 Exp number of AAs in TMHs: 21.08393

# WP_011013739.1 Exp number, first 60 AAs: 0.34162

# WP_011013739.1 Total prob of N-in: 0.03842

WP_011013739.1 TMHMM2.0 outside 1 376

WP_011013739.1 TMHMM2.0 TMhelix 377 396

WP_011013739.1 TMHMM2.0 inside 397 414


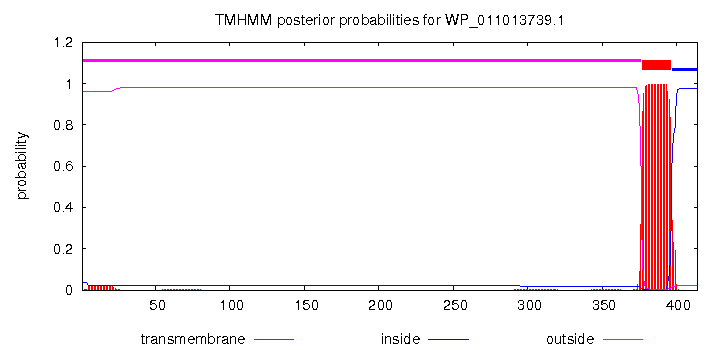


# [plot](http://www.cbs.dtu.dk/services/TMHMM-2.0/tmp/TMHMM_7933/WP_011013739.1.eps) in postscript, [script](http://www.cbs.dtu.dk/services/TMHMM-2.0/tmp/TMHMM_7933/WP_011013739.1.gnuplot) for making the plot in gnuplot, [data](http://www.cbs.dtu.dk/services/TMHMM-2.0/tmp/TMHMM_7933/WP_011013739.1.plp) for plot

gene complement(586396..587640)

/gene="mycP"

/locus_tag="CGL_RS02890"

/old_locus_tag="NCgl0550"

CDS complement(586396..587640)

/gene="mycP"

/locus_tag="CGL_RS02890"

/old_locus_tag="NCgl0550"

/inference="COORDINATES: similar to AA

sequence:RefSeq:WP_003854454.1"

/note="Derived by automated computational analysis using

gene prediction method: Protein Homology."

/codon_start=1

/transl_table=11

/product="type VII secretion-associated serine protease

mycosin"

/protein_id="WP_011013739.1"

/translation="MRRLIAVSLAALFMLASTPATRAQEVEALACPEVAIADPSSAVLDEHLSQSLSQAHQLATGAGVMVAVIDTGVSLHPRLPHLIPGGDFVGAHQSPDVPGELIDCDGHGTIVAGIIASQGNPGTGWPYDGSSDPYIGVAPDSGIISIKQTSSYVRTREDSNVGTLSTLAESIHRALDSGAHVINISVVSCLPQSPDEAASFQPLTDALNRAELQGVIVVAAAGNLGQDCPVGSTVYPAHSDTVLSVSARFDSHTLAEYSMPGNQQILSAPSHIQAGLSPRGDGFASHMITTAGESPFEGTSFAAPVVSATAALLRQHFPFATPYEIRARIFNSIDPARGAIDPYLALTQEIYPTTPLVHEIALSVPTPPDDSPRERGILVTAIIVGLLAVLAVLMGLRRIHHHSAFQKASSSVIT"

2

# WP_011013799.1 Length: 234

# WP_011013799.1 Number of predicted TMHs: 1

# WP_011013799.1 Exp number of AAs in TMHs: 25.20672

# WP_011013799.1 Exp number, first 60 AAs: 3.518

# WP_011013799.1 Total prob of N-in: 0.18043

WP_011013799.1 TMHMM2.0 outside 1 200

WP_011013799.1 TMHMM2.0 TMhelix 201 223

WP_011013799.1 TMHMM2.0 inside 224 234


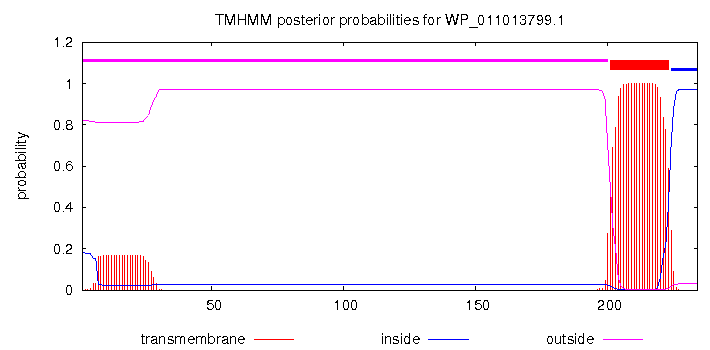


# [plot](http://www.cbs.dtu.dk/services/TMHMM-2.0/tmp/TMHMM_7933/WP_011013799.1.eps) in postscript, [script](http://www.cbs.dtu.dk/services/TMHMM-2.0/tmp/TMHMM_7933/WP_011013799.1.gnuplot) for making the plot in gnuplot, [data](http://www.cbs.dtu.dk/services/TMHMM-2.0/tmp/TMHMM_7933/WP_011013799.1.plp) for plot

gene complement(677044..677748)

/locus_tag="CGL_RS03310"

/old_locus_tag="NCgl0633"

CDS complement(677044..677748)

/locus_tag="CGL_RS03310"

/old_locus_tag="NCgl0633"

/inference="COORDINATES: similar to AA

sequence:RefSeq:**WP_011013799.1**"

/note="Derived by automated computational analysis using

gene prediction method: Protein Homology."

/codon_start=1

/transl_table=11

/product="hypothetical protein"

/protein_id="WP_011013799.1"

/translation="MRFSRVLPALLITTAVSIPTASAATLTADTDKELCIASNTDDSAVVTFWNSIEDSVREQRLDELDAQDPGIKAAIESYIAQDDNAPTAAELQVRLDAIESGEGLAMLLPDDPTLADPNAEESFKTEYTYDEAKDIISGFSSDPASDVLSQLQQAATTGTRTAEIRAEVFADRTDDYNESQTALKEDFQNCIDAIDDARPIPLQYILIGGAIALAVIVLGIRAWTNSRKQSKHSQ"

NCgl0633 WP_011013799.1

gene complement(586396..587640)

/gene="mycP"

/locus_tag="CGL_RS02890"

/old_locus_tag="NCgl0550"

CDS complement(586396..587640)

/gene="mycP"

/locus_tag="CGL_RS02890"

/old_locus_tag="NCgl0550"

/inference="COORDINATES: similar to AA

sequence:RefSeq:WP_003854454.1"

/note="Derived by automated computational analysis using

gene prediction method: Protein Homology."

/codon_start=1

/transl_table=11

/product="type VII secretion-associated serine protease

mycosin"

/protein_id="**WP_011013739.1**"

/translation="MRRLIAVSLAALFMLASTPATRAQEVEALACPEVAIADPSSAVL

DEHLSQSLSQAHQLATGAGVMVAVIDTGVSLHPRLPHLIPGGDFVGAHQSPDVPGELI

DCDGHGTIVAGIIASQGNPGTGWPYDGSSDPYIGVAPDSGIISIKQTSSYVRTREDSN

VGTLSTLAESIHRALDSGAHVINISVVSCLPQSPDEAASFQPLTDALNRAELQGVIVV

AAAGNLGQDCPVGSTVYPAHSDTVLSVSARFDSHTLAEYSMPGNQQILSAPSHIQAGL

SPRGDGFASHMITTAGESPFEGTSFAAPVVSATAALLRQHFPFATPYEIRARIFNSID

PARGAIDPYLALTQEIYPTTPLVHEIALSVPTPPDDSPRERGILVTAIIVGLLAVLAVLMGLRRIHHHSAFQKASSSVIT"

3 **WP_003859654.1 @Multi-transmembrane (Lipid modified N-termini)**

gene complement(2326919..2327998)

/locus_tag="CGL_RS10895"

/old_locus_tag="NCgl2115"

CDS complement(2326919..2327998)

/locus_tag="CGL_RS10895"

/old_locus_tag="NCgl2115"

/inference="COORDINATES: similar to AA

sequence:RefSeq:**WP_003859654.1**"

/note="Derived by automated computational analysis using

gene prediction method: Protein Homology."

/codon_start=1

/transl_table=11

/product="cytochrome c oxidase subunit II"

/protein_id="**WP_011014951.1**"

/translation="MEQQNKRGLKRKALLGGVLGLGGLAMAGCEVAPPGGVLGDFLRMGWPDGITPEAVAMGNFWSWVWVAAWIIGIIMWGLFLTAIFAWGAKRAEKRGEGEFPKQLQYNVPLELVLTIVPIIIVMVLFFFTVQTQDKVTALDKNPEVTVDVTAYQWNWKFGYSEIDGSLAPGGQDYQGSDPERQAAAEASKKDPSGDNPIHGNSKSDVSYLEFNRIETLGTTDEIPVMVLPVNTPIEFNLASADVAHSFWVPEFLFKRDAYAHPEANKSQRVFQIEEITEEGAFVGRCAEMCGTYHAMMNFELRVVDRDSFAEYISFRDSNPDATNAQALEHIGQAP

YATSTSPFVSDRTATRDGENTQSNA"

# WP_011014951.1 Length: 359

# WP_011014951.1 Number of predicted TMHs: 3

# WP_011014951.1 Exp number of AAs in TMHs: 68.0102800000000001

# WP_011014951.1 Exp number, first 60 AAs: 22.54847

# WP_011014951.1 Total prob of N-in: 0.99961

# WP_011014951.1 POSSIBLE N-term signal sequence

WP_011014951.1 TMHMM2.0 inside 1 12

WP_011014951.1 TMHMM2.0 TMhelix 13 35

WP_011014951.1 TMHMM2.0 outside 36 64

WP_011014951.1 TMHMM2.0 TMhelix 65 87

WP_011014951.1 TMHMM2.0 inside 88 106

WP_011014951.1 TMHMM2.0 TMhelix 107 129

WP_011014951.1 TMHMM2.0 outside 130 359


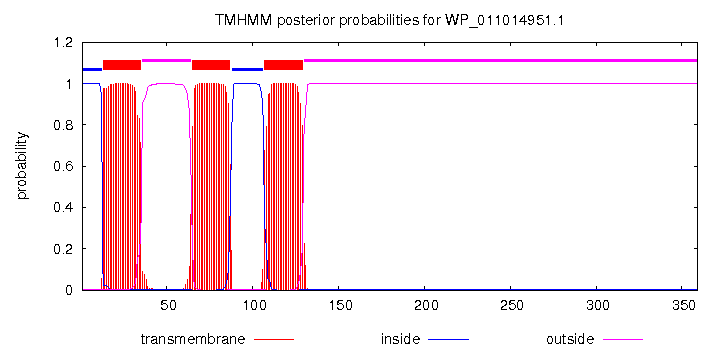


# [plot](http://www.cbs.dtu.dk/services/TMHMM-2.0/tmp/TMHMM_7933/WP_011014951.1.eps) in postscript, [script](http://www.cbs.dtu.dk/services/TMHMM-2.0/tmp/TMHMM_7933/WP_011014951.1.gnuplot) for making the plot in gnuplot, [data](http://www.cbs.dtu.dk/services/TMHMM-2.0/tmp/TMHMM_7933/WP_011014951.1.plp) for plot

4

>WP_011265759.1

# WP_011265759.1 Length: 341

# WP_011265759.1 Number of predicted TMHs: 1

# WP_011265759.1 Exp number of AAs in TMHs: 19.85003

# WP_011265759.1 Exp number, first 60 AAs: 19.82027

# WP_011265759.1 Total prob of N-in: 0.89183

# WP_011265759.1 POSSIBLE N-term signal sequence

WP_011265759.1 TMHMM2.0 inside 1 11

WP_011265759.1 TMHMM2.0 TMhelix 12 34

WP_011265759.1 TMHMM2.0 outside 35 341


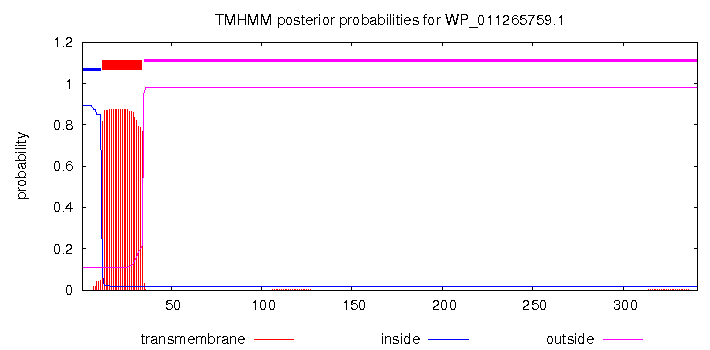


# [plot](http://www.cbs.dtu.dk/services/TMHMM-2.0/tmp/TMHMM_18373/WP_011265759.1.eps) in postscript, [script](http://www.cbs.dtu.dk/services/TMHMM-2.0/tmp/TMHMM_18373/WP_011265759.1.gnuplot) for making the plot in gnuplot, [data](http://www.cbs.dtu.dk/services/TMHMM-2.0/tmp/TMHMM_18373/WP_011265759.1.plp) for plot

5

WP_011014306.1 @Lipid anchored

# WP_011014306.1 Length: 366

# WP_011014306.1 Number of predicted TMHs: 0

# WP_011014306.1 Exp number of AAs in TMHs: 17.80803

# WP_011014306.1 Exp number, first 60 AAs: 17.79535

# WP_011014306.1 Total prob of N-in: 0.73297

# WP_011014306.1 POSSIBLE N-term signal sequence

WP_011014306.1 TMHMM2.0 outside 1 366


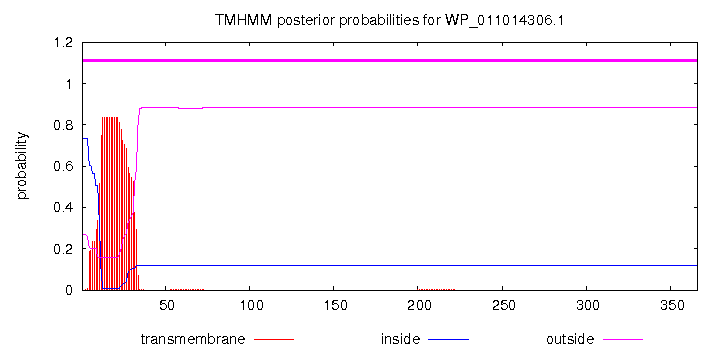


# [plot](http://www.cbs.dtu.dk/services/TMHMM-2.0/tmp/TMHMM_7933/WP_011014306.1.eps) in postscript, [script](http://www.cbs.dtu.dk/services/TMHMM-2.0/tmp/TMHMM_7933/WP_011014306.1.gnuplot) for making the plot in gnuplot, [data](http://www.cbs.dtu.dk/services/TMHMM-2.0/tmp/TMHMM_7933/WP_011014306.1.plp) for plot

gene 1426257..1427357

/locus_tag="CGL_RS06795"

/old_locus_tag="NCgl1307"

CDS 1426257..1427357

/locus_tag="CGL_RS06795"

/old_locus_tag="NCgl1307"

/inference="COORDINATES: similar to AA

sequence:RefSeq:WP_011897187.1"

/note="Derived by automated computational analysis using

gene prediction method: Protein Homology."

/codon_start=1

/transl_table=11

/product="hypothetical protein"

/protein_id="WP_011014306.1"

/translation="MKDYAVHTRGLVSLLSLIFLFVLSGCGGNATTADEAAESDVVTS

SSAPPSKRALDVGEAVEIPGVVLTVNSVTQSDQLMLYSEGSARGSEPREQRNAASGEK

FVSVDTTVKNSSSDPWDLSCGHVLQTWLLEDELDDQQGDQEKKFSPIDNLDQISGNPE

CGVLLEVGTEIEMTWSFTIPDDIEITHFGFSLSDSTSNDLAIISLGGAIETSSAITTT

EVIAPENDTETLLEITPVDCQVGLGPIVTSWSDGTVGGWSQHCQDVHDEVLAGEVAAN

TPVCDGVVCTYPSGATMPDPNAPQIPSDTSGAVCDENQCVYPNGYIARIGDPNVPNYL

KPGNSPWVQGQIDFQNCLDSGKTIEQCREELN"

6

# WP_011014779.1 Length: 295

# WP_011014779.1 Number of predicted TMHs: 0

# WP_011014779.1 Exp number of AAs in TMHs: 11.02134

# WP_011014779.1 Exp number, first 60 AAs: 11.02018

# WP_011014779.1 Total prob of N-in: 0.58364

# WP_011014779.1 POSSIBLE N-term signal sequence

WP_011014779.1 TMHMM2.0 outside 1 295


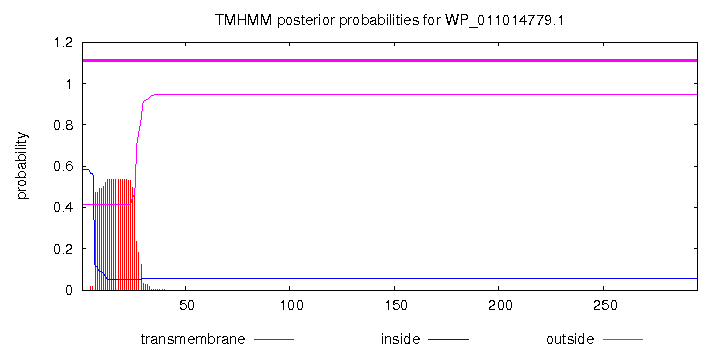


# [plot](http://www.cbs.dtu.dk/services/TMHMM-2.0/tmp/TMHMM_7933/WP_011014779.1.eps) in postscript, [script](http://www.cbs.dtu.dk/services/TMHMM-2.0/tmp/TMHMM_7933/WP_011014779.1.gnuplot) for making the plot in gnuplot, [data](http://www.cbs.dtu.dk/services/TMHMM-2.0/tmp/TMHMM_7933/WP_011014779.1.plp) for plot

gene 2060620..2061507

/locus_tag="CGL_RS09710"

/old_locus_tag="NCgl1876"

CDS 2060620..2061507

/locus_tag="CGL_RS09710"

/old_locus_tag="NCgl1876"

/inference="COORDINATES: similar to AA

sequence:RefSeq:WP_003861643.1"

/note="Derived by automated computational analysis using

gene prediction method: Protein Homology."

/codon_start=1

/transl_table=11

/product="glutamate ABC transporter substrate-binding

protein"

/protein_id="WP_011014779.1"

/translation="MSAKRTFTRIGAILGATALAGVTLTACGDSSGGDGFLAAIENGS

VNVGTKYDQPGLGLRNPDNSMSGLDVDVAEYVVNSIADDKGWDHPTIEWRESPSAQRE

TLIQNGEVDMIAATYSINAGRSESVNFGGPYLLTHQALLVRQDDDRIETLEDLDNGLI

LCSVSGSTPAQKVKDVLPGVQLQEYDTYSSCVEALSQGNVDALTTDATILFGYSQQYE

GDFRVVEMEKDGEPFTDEYYGIGLKKDDQEGTDAINAALERMYADGTFQRLLTENLGE

DSVVVEEGTPGDLSFLDAS"

7

# WP_011265985.1 Length: 536

# WP_011265985.1 Number of predicted TMHs: 0

# WP_011265985.1 Exp number of AAs in TMHs: 14.57839

# WP_011265985.1 Exp number, first 60 AAs: 14.57396

# WP_011265985.1 Total prob of N-in: 0.69370

# WP_011265985.1 POSSIBLE N-term signal sequence

WP_011265985.1 TMHMM2.0 outside 1 536


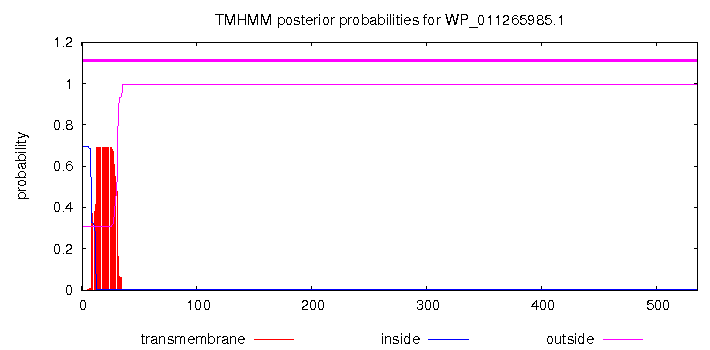


# [plot](http://www.cbs.dtu.dk/services/TMHMM-2.0/tmp/TMHMM_7933/WP_011265985.1.eps) in postscript, [script](http://www.cbs.dtu.dk/services/TMHMM-2.0/tmp/TMHMM_7933/WP_011265985.1.gnuplot) for making the plot in gnuplot, [data](http://www.cbs.dtu.dk/services/TMHMM-2.0/tmp/TMHMM_7933/WP_011265985.1.plp) for plot

gene 2820585..2822195

/locus_tag="CGL_RS13230"

/old_locus_tag="NCgl2562"

CDS 2820585..2822195

/locus_tag="CGL_RS13230"

/old_locus_tag="NCgl2562"

/inference="COORDINATES: similar to AA

sequence:RefSeq:WP_011265985.1"

/note="Derived by automated computational analysis using

gene prediction method: Protein Homology."

/codon_start=1

/transl_table=11

/product="twin-arginine translocation signal

domain-containing protein"

/protein_id="WP_011265985.1"

/translation="MSTTITRRNFLRATGILGVAAGIGATLAACAPDNTGTSGSTSTA

AGTGTANEEGTITAAISYELGTNGYDPMTTTSALTVAANWHTLEGLTEIDPATGEVYA

ALASALPSADATSLDIKLRDGATFHNGDAVTADDVVFSFERVLDPANNSLYASFIPFI

KSVTKKDDTTVTIDLDYATGIISERLAVVKIVPKSVVEADASGFDANPIGSGPYKMTD

NGASKVVKFERNDDYNGPRPARAAKMEWQIIPDASTRTNSLQSGSTMAIDSVPYLSIP

QLEATSTVESVQGFGLLFAMFNCSEGNPFNDVRNRQAFLYALDMDKIVKTGMSDQATP

ATSFVQKEHPNYNQASTVYSLDADKAKALFAETGLTSLNLLCTDHDWVKNCTPLIQES

LAALGINVSFTERKSADVYNTIDGKPEAYDVVIAPGDPSVFGNDPDLLMRWWYAGDVW

TDSRMHWKGSESYDQVQNLLEEGIRATDKAEQQDIWNRTFDVISDNVPLYPLFHRKVP

TAWNSNALVDFKPISLTGLNFSGVATTE"

8

# WP_011013364.1 Length: 551

# WP_011013364.1 Number of predicted TMHs: 2

# WP_011013364.1 Exp number of AAs in TMHs: 45.52837

# WP_011013364.1 Exp number, first 60 AAs: 22.88811

# WP_011013364.1 Total prob of N-in: 0.02712

# WP_011013364.1 POSSIBLE N-term signal sequence

WP_011013364.1 TMHMM2.0 outside 1 22

WP_011013364.1 TMHMM2.0 TMhelix 23 45

WP_011013364.1 TMHMM2.0 inside 46 191

WP_011013364.1 TMHMM2.0 TMhelix 192 214

WP_011013364.1 TMHMM2.0 outside 215 551


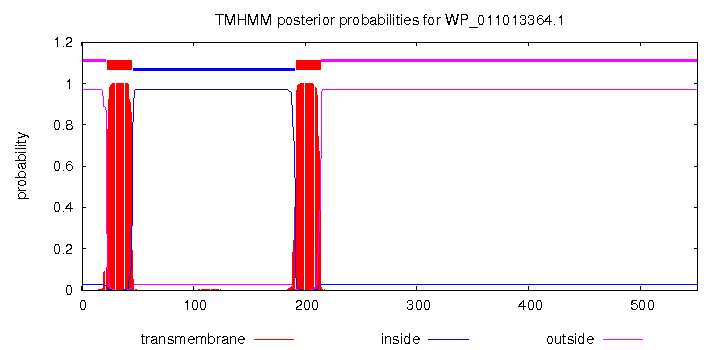


# [plot](http://www.cbs.dtu.dk/services/TMHMM-2.0/tmp/TMHMM_7933/WP_011013364.1.eps) in postscript, [script](http://www.cbs.dtu.dk/services/TMHMM-2.0/tmp/TMHMM_7933/WP_011013364.1.gnuplot) for making the plot in gnuplot, [data](http://www.cbs.dtu.dk/services/TMHMM-2.0/tmp/TMHMM_7933/WP_011013364.1.plp) for plot

gene 70506..72161

/locus_tag="CGL_RS00375"

/old_locus_tag="NCgl0067"

CDS 70506..72161

/locus_tag="CGL_RS00375"

/old_locus_tag="NCgl0067"

/inference="COORDINATES: similar to AA

sequence:RefSeq:WP_011013364.1"

/note="Derived by automated computational analysis using

gene prediction method: Protein Homology."

/codon_start=1

/transl_table=11

/product="sensor histidine kinase"

/protein_id="WP_011013364.1"

/translation="MSVGGSDWKNFKEVDIIRFATRILVIQVATVALVVAICTGIFAV

LMMDQMKTEAEHTALSIGRSVASNPQIREEVALDTQTGANPSAEELADGDIQAVAQAA

NERTGALFVVITDGLGIRLSHPDEERLGEQVSTSFEAAMRGEETMAWETGTLGASARA

KVPIFAPDSSVPVGEVSVGFERDSVYSRLPMFLAALALISVLGILIGVGVAMGMRRRW

ERVTLGLQPEELVTLVQNQTAVIDGIDEGVLALSPNGTIGVHNEQAQSMIGAGPMSGR

TLKELGLDLGLDGVVLHGQHPETVAHNGRILYLDFHPVRRGDQDLGYVVTIRDRTDII

ELSERLDSVRTMTHALRAQRHEFANRIHTATGLIDAGRVHDAAEFLGDISRNGGQSHP

LIGSAHLNEAFLSSFLSTASISASEKGVSLRINSDTLILGTVKDPEDVATILGNLINN

AIDAAVAGEAPRWIELTLMDDADTLVISVADSGPGIPEGVDVFATATQIGDSEDNERT

HGHGIGLKLCRALARSHGGDVWVIDRGTEDGAVFGVKLPGVME"

9

# WP_004567665.1 Length: 141

# WP_004567665.1 Number of predicted TMHs: 3

# WP_004567665.1 Exp number of AAs in TMHs: 71.31589

# WP_004567665.1 Exp number, first 60 AAs: 24.46692

# WP_004567665.1 Total prob of N-in: 0.20597

# WP_004567665.1 POSSIBLE N-term signal sequence

WP_004567665.1 TMHMM2.0 outside 1 26

WP_004567665.1 TMHMM2.0 TMhelix 27 49

WP_004567665.1 TMHMM2.0 inside 50 61

WP_004567665.1 TMHMM2.0 TMhelix 62 84

WP_004567665.1 TMHMM2.0 outside 85 98

WP_004567665.1 TMHMM2.0 TMhelix 99 121

WP_004567665.1 TMHMM2.0 inside 122 141


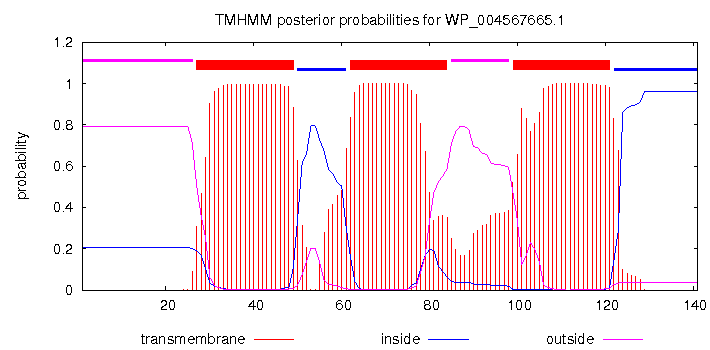


# [plot](http://www.cbs.dtu.dk/services/TMHMM-2.0/tmp/TMHMM_7933/WP_004567665.1.eps) in postscript, [script](http://www.cbs.dtu.dk/services/TMHMM-2.0/tmp/TMHMM_7933/WP_004567665.1.gnuplot) for making the plot in gnuplot, [data](http://www.cbs.dtu.dk/services/TMHMM-2.0/tmp/TMHMM_7933/WP_004567665.1.plp) for plot

gene complement(2513690..2514115)

/locus_tag="CGL_RS11770"

/old_locus_tag="NCgl2291"

CDS complement(2513690..2514115)

/locus_tag="CGL_RS11770"

/old_locus_tag="NCgl2291"

/inference="COORDINATES: similar to AA

sequence:RefSeq:WP_003859287.1"

/note="Derived by automated computational analysis using

gene prediction method: Protein Homology."

/codon_start=1

/transl_table=11

/product="DUF4233 domain-containing protein"

/protein_id="WP_004567665.1"

/translation="MSKREESIEYGPLGKGHDPLKDPMKGIRGVMAGTLVMEAITLGL

VLTVILRVDDGIYWTTFNWVYVSAVAIAHFVAAFLQRFSWSIPMNIVLQVLALAGFFV

HPAMGFAAIIFIIAWAYLFYLRSNLIDRMKRGLLTTQHS"

10

# WP_011014192.1 Length: 467

# WP_011014192.1 Number of predicted TMHs: 4

# WP_011014192.1 Exp number of AAs in TMHs: 90.04242

# WP_011014192.1 Exp number, first 60 AAs: 22.89888

# WP_011014192.1 Total prob of N-in: 0.03175

# WP_011014192.1 POSSIBLE N-term signal sequence

WP_011014192.1 TMHMM2.0 outside 1 3

WP_011014192.1 TMHMM2.0 TMhelix 4 26

WP_011014192.1 TMHMM2.0 inside 27 60

WP_011014192.1 TMHMM2.0 TMhelix 61 83

WP_011014192.1 TMHMM2.0 outside 84 97

WP_011014192.1 TMHMM2.0 TMhelix 98 120

WP_011014192.1 TMHMM2.0 inside 121 139

WP_011014192.1 TMHMM2.0 TMhelix 140 162

WP_011014192.1 TMHMM2.0 outside 163 467


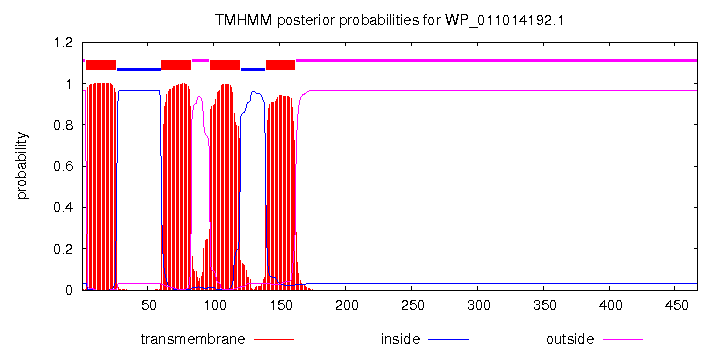


# [plot](http://www.cbs.dtu.dk/services/TMHMM-2.0/tmp/TMHMM_7933/WP_011014192.1.eps) in postscript, [script](http://www.cbs.dtu.dk/services/TMHMM-2.0/tmp/TMHMM_7933/WP_011014192.1.gnuplot) for making the plot in gnuplot, [data](http://www.cbs.dtu.dk/services/TMHMM-2.0/tmp/TMHMM_7933/WP_011014192.1.plp) for plot

gene complement(1257862..1259265)

/locus_tag="CGL_RS05950"

/old_locus_tag="NCgl1147"

CDS complement(1257862..1259265)

/locus_tag="CGL_RS05950"

/old_locus_tag="NCgl1147"

/inference="COORDINATES: similar to AA

sequence:RefSeq:WP_011897093.1"

/note="Derived by automated computational analysis using

gene prediction method: Protein Homology."

/codon_start=1

/transl_table=11

/product="HlyC/CorC family transporter"

/protein_id="WP_011014192.1"

/translation="MLTAVLSLIAGLVVIGVIIVLNGYFVAQEFAYMSVDRNELRALA

DSGDKKARRALSITKRTSFMLSGAQLGITVTGLLVGFVAEPLVGNALGVLLGGVGVPA

AVSISVGTVLALAISTVVQMIFGELFPKNYTLATPLKSALALAPSTTWYLKLAGWLIT

FFDFASNALLRLFRIEPVEDVDSSATAQDLPHIVASSRDSGVLNNSMSLSLDRLLDFP

SHDVGHAMIPRSRVGVVDPETTIAEVKALMRKAHTRYPIIDDNHVPIGVINLIDILGT

DIHGAEISDSAKVTEFMHQPVIVPEFMSLPDVVTELHNREDRLACVIDEYGGFIGIVT

LEDLAEEVLGDINDEHDVFSSEDITETSPDKWLIDGDTPLDEVERAIGYELPEGDYET

ISGLLFDHANALLKTGDVIEIPLDFEPEDYLNNTSPTQRILRITVLEVERNVPVKLAL

ALLEDHPDNHPAPKENR"

11

# WP_011014865.1 Length: 510

# WP_011014865.1 Number of predicted TMHs: 5

# WP_011014865.1 Exp number of AAs in TMHs: 111.87541

# WP_011014865.1 Exp number, first 60 AAs: 35.84029

# WP_011014865.1 Total prob of N-in: 0.98452

# WP_011014865.1 POSSIBLE N-term signal sequence

WP_011014865.1 TMHMM2.0 inside 1 19

WP_011014865.1 TMHMM2.0 TMhelix 20 42

WP_011014865.1 TMHMM2.0 outside 43 46

WP_011014865.1 TMHMM2.0 TMhelix 47 69

WP_011014865.1 TMHMM2.0 inside 70 120

WP_011014865.1 TMHMM2.0 TMhelix 121 143

WP_011014865.1 TMHMM2.0 outside 144 146

WP_011014865.1 TMHMM2.0 TMhelix 147 169

WP_011014865.1 TMHMM2.0 inside 170 239

WP_011014865.1 TMHMM2.0 TMhelix 240 262

WP_011014865.1 TMHMM2.0 outside 263 510


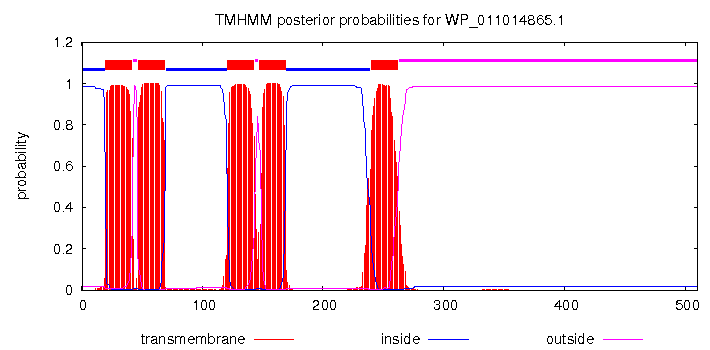


# [plot](http://www.cbs.dtu.dk/services/TMHMM-2.0/tmp/TMHMM_7933/WP_011014865.1.eps) in postscript, [script](http://www.cbs.dtu.dk/services/TMHMM-2.0/tmp/TMHMM_7933/WP_011014865.1.gnuplot) for making the plot in gnuplot, [data](http://www.cbs.dtu.dk/services/TMHMM-2.0/tmp/TMHMM_7933/WP_011014865.1.plp) for plot

gene 2193165..2194697

/locus_tag="CGL_RS10315"

/old_locus_tag="NCgl1998"

CDS 2193165..2194697

/locus_tag="CGL_RS10315"

/old_locus_tag="NCgl1998"

/inference="COORDINATES: similar to AA

sequence:RefSeq:WP_003856383.1"

/note="Derived by automated computational analysis using

gene prediction method: Protein Homology."

/codon_start=1

/transl_table=11

/product="ABC transporter ATP-binding protein"

/protein_id="WP_011014865.1"

/translation="MISRLLQLAKKVWPELGASTLLRLLNQLLTAALIVFPAWVLSRK

PDISLLAVAIIMALIALTAAVCRWGEQVCGHRAAFGLLAHMRVMLYDALVHKGSPSPI

HGSGSIMSVATRDINSIEVFFAHTIGPTVTAVLLSAGGVITLATLDPVAGLIGLLGVL

IAWLIPLIGKQSSSSEATSRGHIAQHLTEDAAGRLEINSHGAQATRLNALEVKEQQLE

QVVTRQGLIVGIRQGAALLWPWISAVLLVALVPHVGIVAAAIILGISPALDAVEGFAR

TMPTALNSAQRYFQIIDAPVAIAEPDEPKPLPKGPLKLRISRVPVSAKGTVSLEVAAG

EHIGIIGSSGSGKSTLAKLILKLAQLRSGTITIGGVDIAEVSSAELRKSVTLVEQKSV

LFRASVLENLRMGNPELSEDEAREALRLASISELPLDADALRLSGGQQQRLCLARALA

RTPQVLIVDEATSHQDALNQADLSQTLATLKDTTVIIIAHRTAALTHVDRIIDLEEIK

NP"

12

# WP_003859459.1 Length: 440

# WP_003859459.1 Number of predicted TMHs: 4

# WP_003859459.1 Exp number of AAs in TMHs: 88.9375700000000001

# WP_003859459.1 Exp number, first 60 AAs: 22.39455

# WP_003859459.1 Total prob of N-in: 0.02419

# WP_003859459.1 POSSIBLE N-term signal sequence

WP_003859459.1 TMHMM2.0 outside 1 9

WP_003859459.1 TMHMM2.0 TMhelix 10 32

WP_003859459.1 TMHMM2.0 inside 33 64

WP_003859459.1 TMHMM2.0 TMhelix 65 87

WP_003859459.1 TMHMM2.0 outside 88 91

WP_003859459.1 TMHMM2.0 TMhelix 92 114

WP_003859459.1 TMHMM2.0 inside 115 126

WP_003859459.1 TMHMM2.0 TMhelix 127 149

WP_003859459.1 TMHMM2.0 outside 150 440


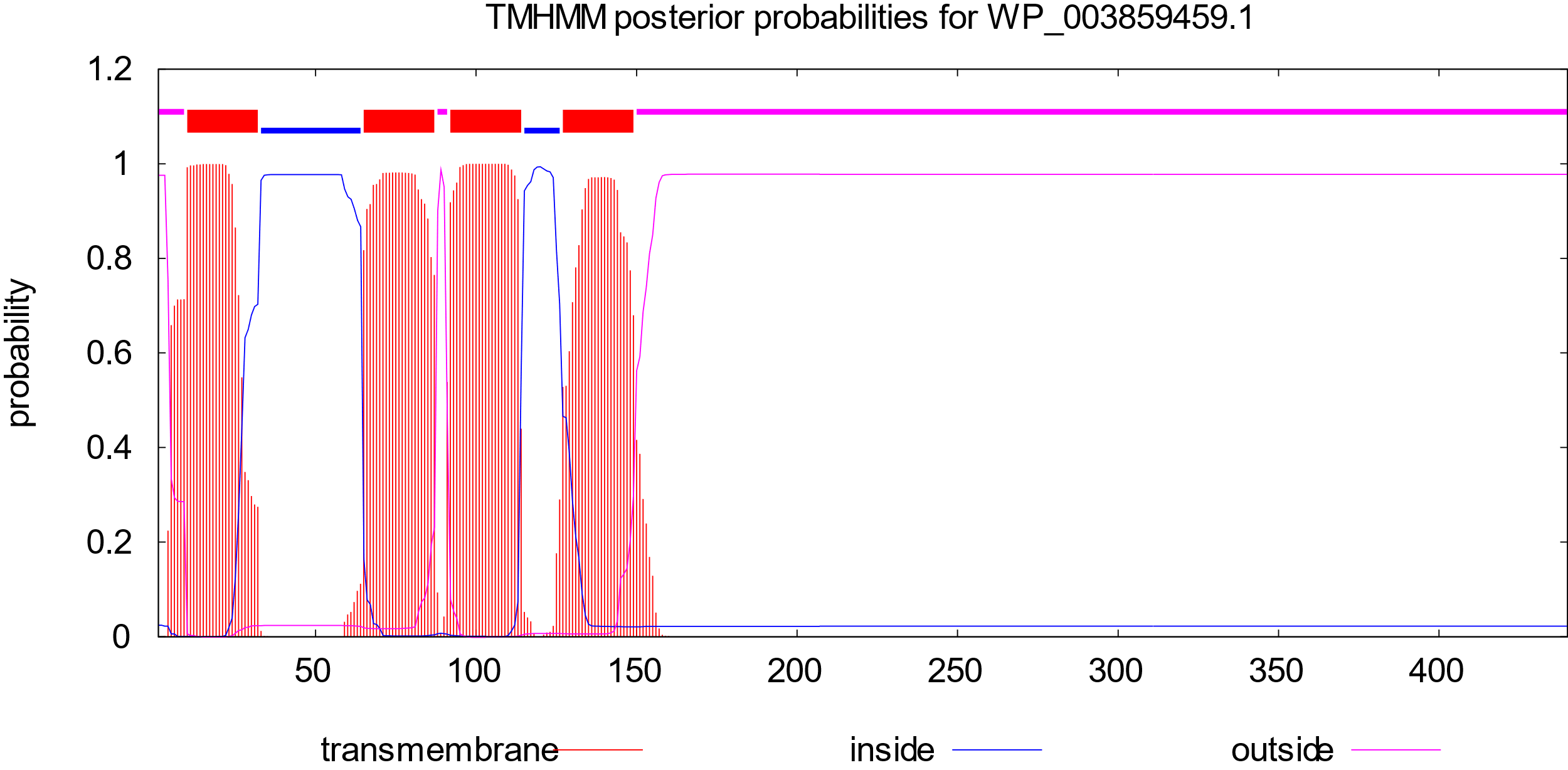


# [plot](http://www.cbs.dtu.dk/services/TMHMM-2.0/tmp/TMHMM_7933/WP_003859459.1.eps) in postscript, [script](http://www.cbs.dtu.dk/services/TMHMM-2.0/tmp/TMHMM_7933/WP_003859459.1.gnuplot) for making the plot in gnuplot, [data](http://www.cbs.dtu.dk/services/TMHMM-2.0/tmp/TMHMM_7933/WP_003859459.1.plp) for plot

gene complement(2418988..2420310)

/locus_tag="CGL_RS11335"

/old_locus_tag="NCgl2206"

CDS complement(2418988..2420310)

/locus_tag="CGL_RS11335"

/old_locus_tag="NCgl2206"

/inference="COORDINATES: similar to AA

sequence:RefSeq:WP_003859459.1"

/note="Derived by automated computational analysis using

gene prediction method: Protein Homology."

/codon_start=1

/transl_table=11

/product="HlyC/CorC family transporter"

/protein_id="WP_003859459.1"

/translation="MESSVIWLSIATVVALLFSGLLGAVESALSSVSRARVEQMLKDE

ASGSASLLRVIDERALHINMLIMLRTLLDASAAVFAGAIAVNVMDSWAWGIVLAIVVV

SLLTFAVVGVFGRTVGRKNPYSVMLRSAVVLSGLAKILGPIARGLIWIGNIIAPGPGF

RNGPYATEVELREMVDIAQEHGIVEIEERRMIQSVFDLASTTVRQVMVPRPEMIWIES

GKTAGQATALCVRSGHSRIPVIGENVDDIIGIVYLKDLVQKTYYATDGGKSVLVDEVM

REATFVPDSKSLDALLQEMQEDHKHIAILVDEYGGVAGLISIEDILEEIVGEIADEYD

AREVAPIEKIGDRTYRVVSRLSLEDLKDHIEEELDLEIEFGDEIEDQVDTVGGLIAFE

LGRVPLPGATVETCGLKLTAEGAKNRRGRLRMHSAVVEVGEPSEDNEG"

13

# WP_003863539.1 Length: 353

# WP_003863539.1 Number of predicted TMHs: 3

# WP_003863539.1 Exp number of AAs in TMHs: 62.6236199999999999

# WP_003863539.1 Exp number, first 60 AAs: 22.60486

# WP_003863539.1 Total prob of N-in: 0.95718

# WP_003863539.1 POSSIBLE N-term signal sequence

WP_003863539.1 TMHMM2.0 inside 1 32

WP_003863539.1 TMHMM2.0 TMhelix 33 55

WP_003863539.1 TMHMM2.0 outside 56 64

WP_003863539.1 TMHMM2.0 TMhelix 65 84

WP_003863539.1 TMHMM2.0 inside 85 96

WP_003863539.1 TMHMM2.0 TMhelix 97 119

WP_003863539.1 TMHMM2.0 outside 120 353


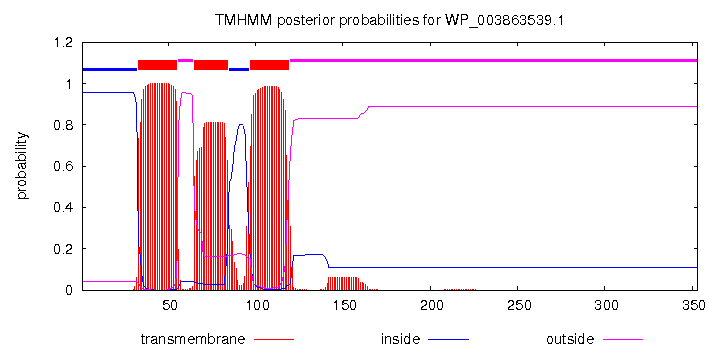


# [plot](http://www.cbs.dtu.dk/services/TMHMM-2.0/tmp/TMHMM_7933/WP_003863539.1.eps) in postscript, [script](http://www.cbs.dtu.dk/services/TMHMM-2.0/tmp/TMHMM_7933/WP_003863539.1.gnuplot) for making the plot in gnuplot, [data](http://www.cbs.dtu.dk/services/TMHMM-2.0/tmp/TMHMM_7933/WP_003863539.1.plp) for plot

gene 817465..818526

/locus_tag="CGL_RS03875"

/old_locus_tag="NCgl0743"

CDS 817465..818526

/locus_tag="CGL_RS03875"

/old_locus_tag="NCgl0743"

/inference="COORDINATES: similar to AA

sequence:RefSeq:WP_003863539.1"

/note="Derived by automated computational analysis using

gene prediction method: Protein Homology."

/codon_start=1

/transl_table=11

/product="potassium channel family protein"

/protein_id="WP_003863539.1"

/translation="MGRMKNDGELADLPDHALLSIIRIPQAAKRSPWALILTRIGYAM

VLLVIVTMVVYFDRNGYSEDLTFIDALYYSTVSLTTVGYGDITPVTQSARLINIIVLT

PARIGFLILLVGTTLSVLTEESRRALQIQRWRKRMRNHTVVVGYGTKGRSAVAALLAD

GVPANQIVVIDTDQVSLDAANNSGLVTVKGSATKADVLRLAGVSRARAVVVAPNLDDT

AVLVTLSVREIAPQAMIVASVRESENQHLLEQSGADSVVISSETAGRMLGLATVTPSV

VEMMEDLLSPDEGFSVAERLVGEDEIGSNPRHLADIVLGVVRSGELYRIDSPEAETVE

PGDRLLYVRRVFSEEVNDK"

14

# WP_003856281.1 Length: 554

# WP_003856281.1 Number of predicted TMHs: 1

# WP_003856281.1 Exp number of AAs in TMHs: 18.32236

# WP_003856281.1 Exp number, first 60 AAs: 18.31511

# WP_003856281.1 Total prob of N-in: 0.81366

# WP_003856281.1 POSSIBLE N-term signal sequence

WP_003856281.1 TMHMM2.0 inside 1 12

WP_003856281.1 TMHMM2.0 TMhelix 13 35

WP_003856281.1 TMHMM2.0 outside 36 554


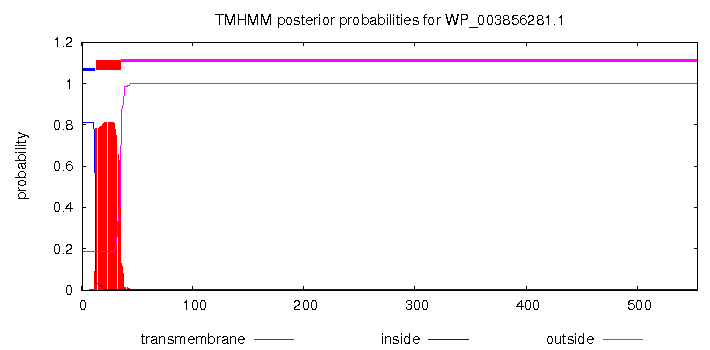


# [plot](http://www.cbs.dtu.dk/services/TMHMM-2.0/tmp/TMHMM_7933/WP_003856281.1.eps) in postscript, [script](http://www.cbs.dtu.dk/services/TMHMM-2.0/tmp/TMHMM_7933/WP_003856281.1.gnuplot) for making the plot in gnuplot, [data](http://www.cbs.dtu.dk/services/TMHMM-2.0/tmp/TMHMM_7933/WP_003856281.1.plp) for plot

gene 1493513..1495177

/locus_tag="CGL_RS07090"

/old_locus_tag="NCgl1362"

CDS 1493513..1495177

/locus_tag="CGL_RS07090"

/old_locus_tag="NCgl1362"

/EC_number="6.3.4.2"

/inference="COORDINATES: similar to AA

sequence:RefSeq:WP_003856281.1"

/note="Derived by automated computational analysis using

gene prediction method: Protein Homology."

/codon_start=1

/transl_table=11

/product="CTP synthase"

/protein_id="WP_003856281.1"

/translation="MTSSRKVRPTKHIFVTGGVVSSLGKGLTAASLGQLLIARGLSVT

MQKLDPYLNVDPGTMNPFEHGEVFVTEDGAETDLDLGHYERFLDRNLGLNANVTTGKV

YSTVIAKERRGEYLGKTVQVIPHITDEIKARILSMGEPDAHGNAPDVVISEVGGTVGD

IESQPFLEAARQVRHEIGRENCFFIHCSLVPYLATSGELKTKPTQHSVAELRGIGILP

DALVLRCDREVPQGLKDKIAMMCDVDYEGVVSCPDSSSIYNIPDVLYREHLDTFIIRR

LGLPFRDVDWSTWHDLLERVNNPRHELTVGIVGKYIDLPDAYLSVVEAVRAAGYANWT

RTNIKWITSDDCETPSGAMKALSGLDAIVVPGGFGIRGIEGKIGAITFAREHKIPLLG

LCLGLQCTVIEAARQAGLEQASSTEFDPAATQPVIATMEEQKAAVSGEADLGGTMRLG

AYPATLEEGSLVAELYGTTEVSERHRHRYEVNNAYRAQIAEGSDLVFSGTSPDGHLVE

FVEYPKEVHPYLVATQAHPEYKSRPTHAHPLFYGLVKTALELRVHP"

14

# WP_011013342.1 Length: 474

# WP_011013342.1 Number of predicted TMHs: 1

# WP_011013342.1 Exp number of AAs in TMHs: 22.48962

# WP_011013342.1 Exp number, first 60 AAs: 22.4317

# WP_011013342.1 Total prob of N-in: 0.99873

# WP_011013342.1 POSSIBLE N-term signal sequence

WP_011013342.1 TMHMM2.0 inside 1 6

WP_011013342.1 TMHMM2.0 TMhelix 7 29

WP_011013342.1 TMHMM2.0 outside 30 474


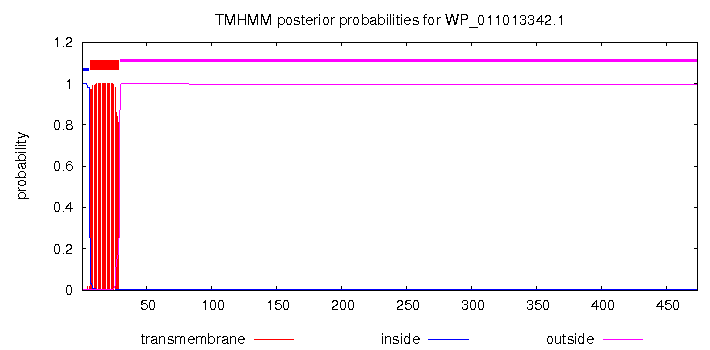


# [plot](http://www.cbs.dtu.dk/services/TMHMM-2.0/tmp/TMHMM_7933/WP_011013342.1.eps) in postscript, [script](http://www.cbs.dtu.dk/services/TMHMM-2.0/tmp/TMHMM_7933/WP_011013342.1.gnuplot) for making the plot in gnuplot, [data](http://www.cbs.dtu.dk/services/TMHMM-2.0/tmp/TMHMM_7933/WP_011013342.1.plp) for plot

gene complement(43923..45347)

/locus_tag="CGL_RS00245"

/old_locus_tag="NCgl0042"

CDS complement(43923..45347)

/locus_tag="CGL_RS00245"

/old_locus_tag="NCgl0042"

/inference="COORDINATES: similar to AA

sequence:RefSeq:WP_015439388.1"

/note="Derived by automated computational analysis using

gene prediction method: Protein Homology."

/codon_start=1

/transl_table=11

/product="penicillin-binding protein 2"

/protein_id="WP_011013342.1"

/translation="MNRSIRITSLFSLLLILVLVANLTWIQAFRDDDLAQNPLNARGF

LEAKSTPRGQISTGGQVLAESSQDDQGFYQRSYITNPTAYAPVVGYLSDVYGAAGLEL

GYNSILNGSDSSLFTSQWLDVISGSPTHGANIELTLDPNAQQTAYEQLSQSGYEGAVV

ALRPSTGEVLAMASSPSYDPNQIVDPATAEDAWAEYTSTEGAPLLNHATQESLPPGSI

FKIITTAAALENGYSADSTVTAEAAVTLPGTNTTLTNYGGQTCAGGGTTTLLTAFQLS

CNTAFVETGIDVGADALRASAEDFGVGQTYSLGLDNVPGGLGEIPDDAALGQSSIGQR

DVQMNVLQAAVMAGTVSNGGVRMEPYLVSRVTGQDLSELSTHKPKSVGGVEPEIAEQL

KTLMEASERNTSGYTGIQIASKTGTAEHGDENTPPHTWYVAFNNDIAVAVLVKDGGGF

GTSATGGQVAAPIGRAVLQAAGGF"

15

# WP_011013540.1 Length: 186

# WP_011013540.1 Number of predicted TMHs: 1

# WP_011013540.1 Exp number of AAs in TMHs: 23.50677

# WP_011013540.1 Exp number, first 60 AAs: 21.93674

# WP_011013540.1 Total prob of N-in: 0.98569

# WP_011013540.1 POSSIBLE N-term signal sequence

WP_011013540.1 TMHMM2.0 inside 1 6

WP_011013540.1 TMHMM2.0 TMhelix 7 29

WP_011013540.1 TMHMM2.0 outside 30 186


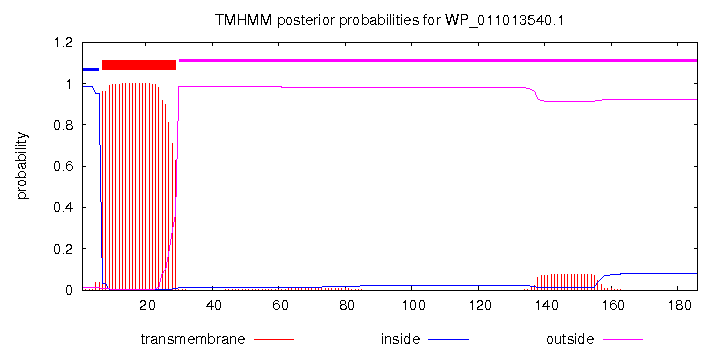


# [plot](http://www.cbs.dtu.dk/services/TMHMM-2.0/tmp/TMHMM_7933/WP_011013540.1.eps) in postscript, [script](http://www.cbs.dtu.dk/services/TMHMM-2.0/tmp/TMHMM_7933/WP_011013540.1.gnuplot) for making the plot in gnuplot, [data](http://www.cbs.dtu.dk/services/TMHMM-2.0/tmp/TMHMM_7933/WP_011013540.1.plp) for plot

gene 308745..309305

/locus_tag="CGL_RS01540"

/old_locus_tag="NCgl0289"

CDS 308745..309305

/locus_tag="CGL_RS01540"

/old_locus_tag="NCgl0289"

/inference="COORDINATES: similar to AA

sequence:RefSeq:WP_003863302.1"

/note="Derived by automated computational analysis using

gene prediction method: Protein Homology."

/codon_start=1

/transl_table=11

/product="TlpA family protein disulfide reductase"

/protein_id="WP_011013540.1"

/translation="MTSSAKWSIVGVVAILAVIVALIPQLVGGESAEEAQGETSTSKI

TTRPDCVASGAAGVDLPCLGGANGVGNELATVVNLWAWWCEPCRAELPIFDEFATTHP

ELNVIGVHADQNAANGAALLEDLGVNLASYQDDSNLFAGTLGLPGVVPITIVVSPDGN

VVDTFPQPFETIDDLETAVAGALQNA"

16

# WP_011014270.1 Length: 60

# WP_011014270.1 Number of predicted TMHs: 1

# WP_011014270.1 Exp number of AAs in TMHs: 21.67789

# WP_011014270.1 Exp number, first 60 AAs: 21.67789

# WP_011014270.1 Total prob of N-in: 0.96355

# WP_011014270.1 POSSIBLE N-term signal sequence

WP_011014270.1 TMHMM2.0 inside 1 8

WP_011014270.1 TMHMM2.0 TMhelix 9 31

WP_011014270.1 TMHMM2.0 outside 32 60


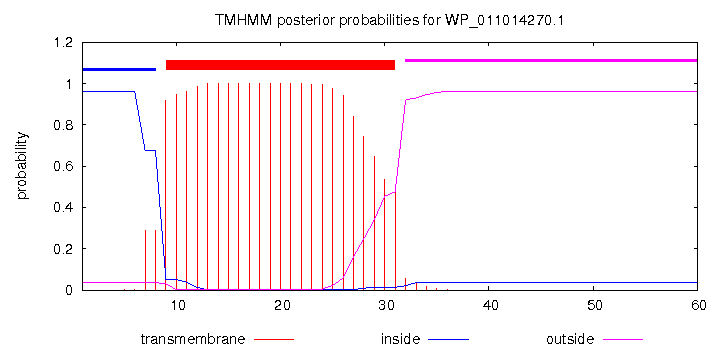


# [plot](http://www.cbs.dtu.dk/services/TMHMM-2.0/tmp/TMHMM_7933/WP_011014270.1.eps) in postscript, [script](http://www.cbs.dtu.dk/services/TMHMM-2.0/tmp/TMHMM_7933/WP_011014270.1.gnuplot) for making the plot in gnuplot, [data](http://www.cbs.dtu.dk/services/TMHMM-2.0/tmp/TMHMM_7933/WP_011014270.1.plp) for plot

gene complement(1365214..1365396)

/locus_tag="CGL_RS06465"

/old_locus_tag="NCgl1250"

CDS complement(1365214..1365396)

/locus_tag="CGL_RS06465"

/old_locus_tag="NCgl1250"

/inference="COORDINATES: similar to AA

sequence:RefSeq:WP_011897149.1"

/note="Derived by automated computational analysis using

gene prediction method: Protein Homology."

/codon_start=1

/transl_table=11

/product="SRPBCC family protein"

/protein_id="WP_011014270.1"

/translation="MKTKKQSRILFIALGLVIALPALSFGLSQLLPATTTREITIDAQ

PDQVWEVLSDLEPFPQ"

17

# WP_011015453.1 Length: 309

# WP_011015453.1 Number of predicted TMHs: 1

# WP_011015453.1 Exp number of AAs in TMHs: 20.74758

# WP_011015453.1 Exp number, first 60 AAs: 20.73199

# WP_011015453.1 Total prob of N-in: 0.97932

# WP_011015453.1 POSSIBLE N-term signal sequence

WP_011015453.1 TMHMM2.0 inside 1 4

WP_011015453.1 TMHMM2.0 TMhelix 5 27

WP_011015453.1 TMHMM2.0 outside 28 309


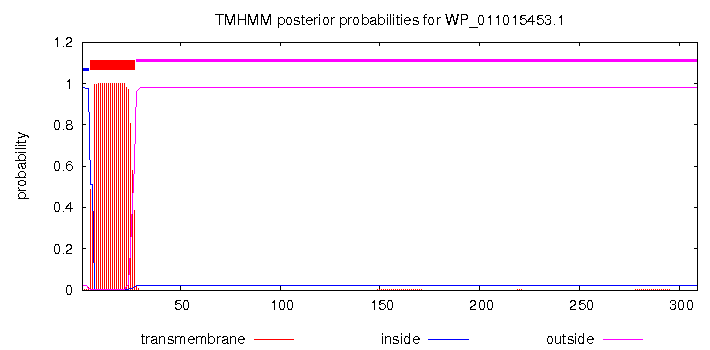


# [plot](http://www.cbs.dtu.dk/services/TMHMM-2.0/tmp/TMHMM_7933/WP_011015453.1.eps) in postscript, [script](http://www.cbs.dtu.dk/services/TMHMM-2.0/tmp/TMHMM_7933/WP_011015453.1.gnuplot) for making the plot in gnuplot, [data](http://www.cbs.dtu.dk/services/TMHMM-2.0/tmp/TMHMM_7933/WP_011015453.1.plp) for plot

gene complement(3070212..3071141)

/locus_tag="CGL_RS14315"

/old_locus_tag="NCgl2775"

CDS complement(3070212..3071141)

/locus_tag="CGL_RS14315"

/old_locus_tag="NCgl2775"

/inference="COORDINATES: similar to AA

sequence:RefSeq:WP_011898015.1"

/note="Derived by automated computational analysis using

gene prediction method: Protein Homology."

/codon_start=1

/transl_table=11

/product="cutinase family protein"

/protein_id="WP_011015453.1"

/translation="MRKTITVIAVLIVLALIGVGIVQYVNTSDDSDFIGQPGEPTGTE

TTEPPVQPDWCPAVEVIAAPGTWESAANDDPINPTANPLSFMLSITQPLQERYSADDV

KVWTLPYTAQFRNINSQNEMSYDDSRNEGTAKMNEELINTHNECPATEFIIVGFSQGA

VIAGDVAAQIGSEQGVIPADSVRGVALIADGRREPGVGQFPGTFVDGIGAEVTLQPLN

LLVQPIVPGATMRGGRAGGFGVLNDRVQDICAPNDAICDAPVNVGNALDRALAMVSAN

GVHALYATNPDVFPGTTTNAWVVDWATNLIDNG"

18

# WP_003853779.1 Length: 137

# WP_003853779.1 Number of predicted TMHs: 1

# WP_003853779.1 Exp number of AAs in TMHs: 21.1903

# WP_003853779.1 Exp number, first 60 AAs: 21.14386

# WP_003853779.1 Total prob of N-in: 0.91031

# WP_003853779.1 POSSIBLE N-term signal sequence

WP_003853779.1 TMHMM2.0 inside 1 4

WP_003853779.1 TMHMM2.0 TMhelix 5 27

WP_003853779.1 TMHMM2.0 outside 28 137


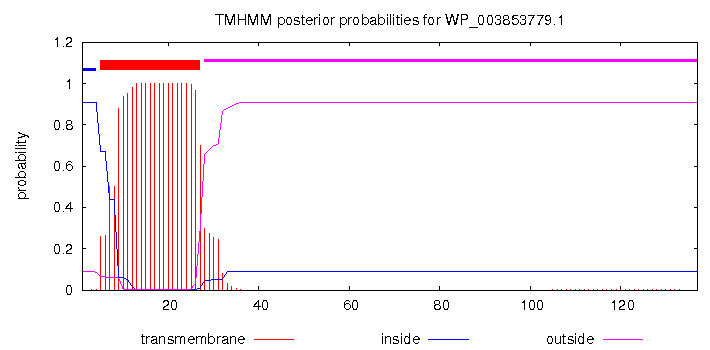


# [plot](http://www.cbs.dtu.dk/services/TMHMM-2.0/tmp/TMHMM_7933/WP_003853779.1.eps) in postscript, [script](http://www.cbs.dtu.dk/services/TMHMM-2.0/tmp/TMHMM_7933/WP_003853779.1.gnuplot) for making the plot in gnuplot, [data](http://www.cbs.dtu.dk/services/TMHMM-2.0/tmp/TMHMM_7933/WP_003853779.1.plp) for plot

gene complement(2875868..2876281)

/locus_tag="CGL_RS13475"

/old_locus_tag="NCgl2610"

CDS complement(2875868..2876281)

/locus_tag="CGL_RS13475"

/old_locus_tag="NCgl2610"

/inference="COORDINATES: similar to AA

sequence:RefSeq:WP_011897913.1"

/note="Derived by automated computational analysis using

gene prediction method: Protein Homology."

/codon_start=1

/transl_table=11

/product="DUF4247 domain-containing protein"

/protein_id="WP_003853779.1"

/translation="MSSRNYRSIGFILLFLAVLCLFAAVFAKPAVGSQVSDRWPGNNG

TYSCAGESGVVDEIVNMSTPTDRATDPATGDTYLRYSKNLIIISGEGTPECTITVEGL

DRVNSGAFIWLGGGFGPSSPSSSSGGSSGSGGGVK"

@Secretory (released) (with CS)

19

# WP_011013420.1 Length: 163

# WP_011013420.1 Number of predicted TMHs: 1

# WP_011013420.1 Exp number of AAs in TMHs: 22.57328

# WP_011013420.1 Exp number, first 60 AAs: 22.17864

# WP_011013420.1 Total prob of N-in: 0.97599

# WP_011013420.1 POSSIBLE N-term signal sequence

WP_011013420.1 TMHMM2.0 inside 1 6

WP_011013420.1 TMHMM2.0 TMhelix 7 29

WP_011013420.1 TMHMM2.0 outside 30 163


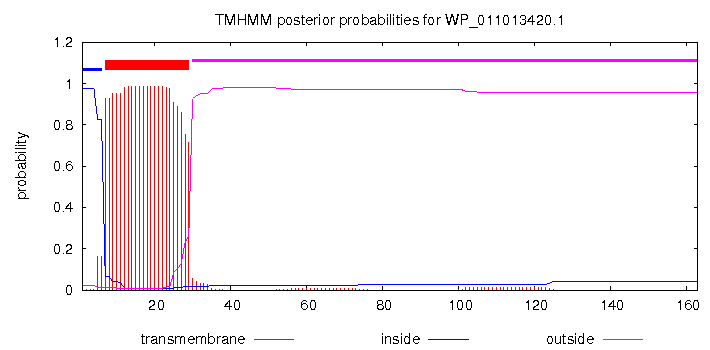


# [plot](http://www.cbs.dtu.dk/services/TMHMM-2.0/tmp/TMHMM_7933/WP_011013420.1.eps) in postscript, [script](http://www.cbs.dtu.dk/services/TMHMM-2.0/tmp/TMHMM_7933/WP_011013420.1.gnuplot) for making the plot in gnuplot, [data](http://www.cbs.dtu.dk/services/TMHMM-2.0/tmp/TMHMM_7933/WP_011013420.1.plp) for plot

gene complement(150963..151454)

/locus_tag="CGL_RS00735"

/old_locus_tag="NCgl0136"

CDS complement(150963..151454)

/locus_tag="CGL_RS00735"

/old_locus_tag="NCgl0136"

/inference="COORDINATES: similar to AA

sequence:RefSeq:WP_003857177.1"

/note="Derived by automated computational analysis using

gene prediction method: Protein Homology."

/codon_start=1

/transl_table=11

/product="hypothetical protein"

/protein_id="WP_011013420.1"

/translation="MRNQTIAAVAALVLLTAATPAIAATPATAGNGLYSIDMGDEQKL

TCVLFDEPSTEAHVVASCAATFPVTWKLLDGAHEQAAKLEITQAQDGELSVTASKQPL

ITTMIAPTSITKPITVNRLVVVPGENEVRFYATDPDVLPVLITPDSYEVLTDSAAKVK

ATL"

20

# WP_011013818.1 Length: 162

# WP_011013818.1 Number of predicted TMHs: 1

# WP_011013818.1 Exp number of AAs in TMHs: 21.26988

# WP_011013818.1 Exp number, first 60 AAs: 21.25332

# WP_011013818.1 Total prob of N-in: 0.95903

# WP_011013818.1 POSSIBLE N-term signal sequence

WP_011013818.1 TMHMM2.0 inside 1 6

WP_011013818.1 TMHMM2.0 TMhelix 7 29

WP_011013818.1 TMHMM2.0 outside 30 162


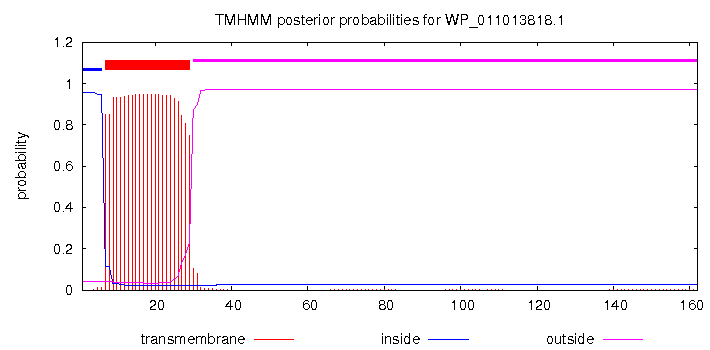


# [plot](http://www.cbs.dtu.dk/services/TMHMM-2.0/tmp/TMHMM_7933/WP_011013818.1.eps) in postscript, [script](http://www.cbs.dtu.dk/services/TMHMM-2.0/tmp/TMHMM_7933/WP_011013818.1.gnuplot) for making the plot in gnuplot, [data](http://www.cbs.dtu.dk/services/TMHMM-2.0/tmp/TMHMM_7933/WP_011013818.1.plp) for plot

gene 709793..710281

/locus_tag="CGL_RS03450"

/old_locus_tag="NCgl0661"

CDS 709793..710281

/locus_tag="CGL_RS03450"

/old_locus_tag="NCgl0661"

/inference="COORDINATES: similar to AA

sequence:RefSeq:WP_003858282.1"

/note="Derived by automated computational analysis using

gene prediction method: Protein Homology."

/codon_start=1

/transl_table=11

/product="CAP domain-containing protein"

/protein_id="WP_011013818.1"

/translation="MKKAMRAAIGLAVSTAMTFGIAPSAHAFTALSSNIFAPPARNTE

NANGDVSQVELEVFALVNQHRIAHGVAPLAMNESLNSGSKSWSYTMSRTGNFVHSSGG

NYGENIYWASNIRPASLIFESWKNSPGHNRNMLDTRYSQIGVGVVYDSSGQTWATTQF

YF"

21

# WP_011013866.1 Length: 261

# WP_011013866.1 Number of predicted TMHs: 1

# WP_011013866.1 Exp number of AAs in TMHs: 21.8299

# WP_011013866.1 Exp number, first 60 AAs: 21.8299

# WP_011013866.1 Total prob of N-in: 0.99769

# WP_011013866.1 POSSIBLE N-term signal sequence

WP_011013866.1 TMHMM2.0 inside 1 6

WP_011013866.1 TMHMM2.0 TMhelix 7 29

WP_011013866.1 TMHMM2.0 outside 30 261


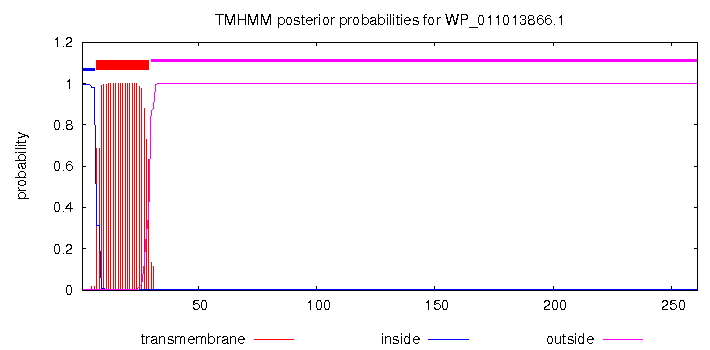


# [plot](http://www.cbs.dtu.dk/services/TMHMM-2.0/tmp/TMHMM_7933/WP_011013866.1.eps) in postscript, [script](http://www.cbs.dtu.dk/services/TMHMM-2.0/tmp/TMHMM_7933/WP_011013866.1.gnuplot) for making the plot in gnuplot, [data](http://www.cbs.dtu.dk/services/TMHMM-2.0/tmp/TMHMM_7933/WP_011013866.1.plp) for plot

gene complement(787167..787952)

/locus_tag="CGL_RS03735"

/old_locus_tag="NCgl0717"

CDS complement(787167..787952)

/locus_tag="CGL_RS03735"

/old_locus_tag="NCgl0717"

/inference="COORDINATES: similar to AA

sequence:RefSeq:WP_011013866.1"

/note="Derived by automated computational analysis using

gene prediction method: Protein Homology."

/codon_start=1

/transl_table=11

/product="hypothetical protein"

/protein_id="WP_011013866.1"

/translation="MKTETRRALVFIVAGCLAATALGFMVWQMSSPSRPTSDIATSTT

TSTTQTQARYDSPGNTETKEAEPDLENQTLAPINTEDPYLPPNAFVRPDNGRSSGLTP

SGSSPTTTSRVSSPSSAGSASPTQITSRSNEPSEPGDESTAATQPSSPDRPTEPTNPV

DPTGPSEPTEPTDPIETTDPIETTDPVAPSTPPTSDDSTSTPQPDESDTPPTDFVEEP

TAPLNPDQPAGSTTDATPNATPSAPADTTSNSVANSVEPTATS"

22

# WP_011014348.1 Length: 321

# WP_011014348.1 Number of predicted TMHs: 1

# WP_011014348.1 Exp number of AAs in TMHs: 21.3604

# WP_011014348.1 Exp number, first 60 AAs: 21.35867

# WP_011014348.1 Total prob of N-in: 0.99411

# WP_011014348.1 POSSIBLE N-term signal sequence

WP_011014348.1 TMHMM2.0 inside 1 8

WP_011014348.1 TMHMM2.0 TMhelix 9 31

WP_011014348.1 TMHMM2.0 outside 32 321


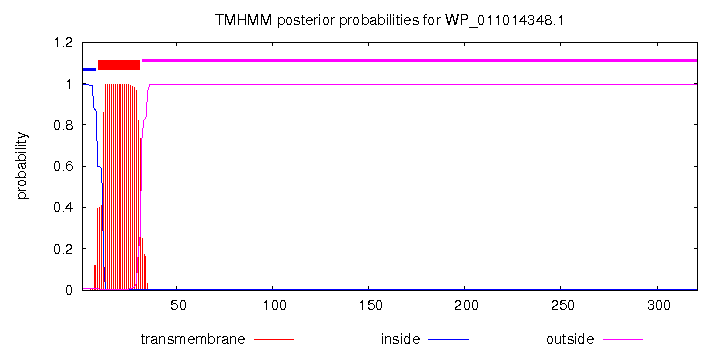


# [plot](http://www.cbs.dtu.dk/services/TMHMM-2.0/tmp/TMHMM_7933/WP_011014348.1.eps) in postscript, [script](http://www.cbs.dtu.dk/services/TMHMM-2.0/tmp/TMHMM_7933/WP_011014348.1.gnuplot) for making the plot in gnuplot, [data](http://www.cbs.dtu.dk/services/TMHMM-2.0/tmp/TMHMM_7933/WP_011014348.1.plp) for plot

gene 1492147..1493112

/locus_tag="CGL_RS07085"

/old_locus_tag="NCgl1361"

CDS 1492147..1493112

/locus_tag="CGL_RS07085"

/old_locus_tag="NCgl1361"

/inference="COORDINATES: similar to AA

sequence:RefSeq:WP_003856283.1"

/note="Derived by automated computational analysis using

gene prediction method: Protein Homology."

/codon_start=1

/transl_table=11

/product="copper transporter"

/protein_id="WP_011014348.1"

/translation="MAKRRGRGAATFAALGFGAAAGIAFGTYVLAPNLPENIDPNAPT

SAELVEAETLAEVNAVQADQADSIIDHIVEDVVAGTLTDRPVLVMRTADAEESDVADV

SWLLQQAGAINAGSITLEENFFSQDGADQLKSIVANTLPAGAQLSETQLDPGTHAGEA

LGAALLLNPETGEPLASTAERGLLLNVLRDNGYISYEDGTILPGQVIVMITGDSDGSG

DGAFAAETQSLFARALDAQGSGVVVAGRIHTAADTGVIGRLRANPDAAENVSTIDSVN

RTWGKMATVLSVREELAGRSGAFGSAASADAASPSLDGTAAAPAQ"

23

# WP_011014599.1 Length: 192

# WP_011014599.1 Number of predicted TMHs: 1

# WP_011014599.1 Exp number of AAs in TMHs: 22.31558

# WP_011014599.1 Exp number, first 60 AAs: 22.31433

# WP_011014599.1 Total prob of N-in: 0.92643

# WP_011014599.1 POSSIBLE N-term signal sequence

WP_011014599.1 TMHMM2.0 inside 1 11

WP_011014599.1 TMHMM2.0 TMhelix 12 34

WP_011014599.1 TMHMM2.0 outside 35 192


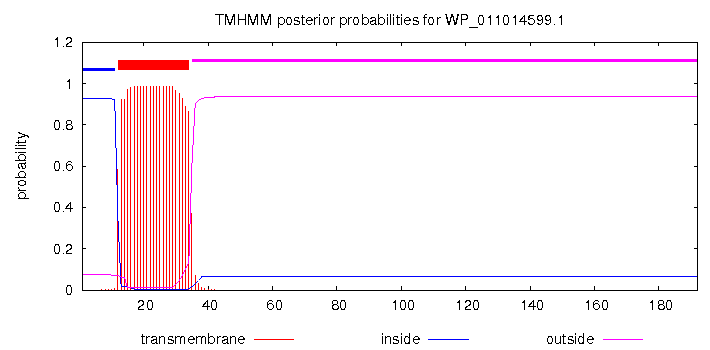


# [plot](http://www.cbs.dtu.dk/services/TMHMM-2.0/tmp/TMHMM_7933/WP_011014599.1.eps) in postscript, [script](http://www.cbs.dtu.dk/services/TMHMM-2.0/tmp/TMHMM_7933/WP_011014599.1.gnuplot) for making the plot in gnuplot, [data](http://www.cbs.dtu.dk/services/TMHMM-2.0/tmp/TMHMM_7933/WP_011014599.1.plp) for plot

gene complement(1850471..1851049)

/locus_tag="CGL_RS08765"

/old_locus_tag="NCgl1682"

CDS complement(1850471..1851049)

/locus_tag="CGL_RS08765"

/old_locus_tag="NCgl1682"

/inference="COORDINATES: similar to AA

sequence:RefSeq:WP_011014599.1"

/note="Derived by automated computational analysis using

gene prediction method: Protein Homology."

/codon_start=1

/transl_table=11

/product="LysM peptidoglycan-binding domain-containing

protein"

/protein_id="WP_011014599.1"

/translation="MAIKGAMPKNRVPGVAAGAFIAAAVIAGGSGVTFLAQGGGDVNT

VAVVEPQDEVKNQVVTETEIVTKVHDPSSSDASDADSNTGTAEGADSDHKEPREHDSA

QEPTAPTDPTLTITGNGDTPVSALDAVAGPARPGTVHVIENGETLSSISQDSGVPVGL

IIDRNKLVDPDLIYAGTPLAIPTEQELAAAIQ"

24

# WP_042383306.1 Length: 211

# WP_042383306.1 Number of predicted TMHs: 1

# WP_042383306.1 Exp number of AAs in TMHs: 19.55342

# WP_042383306.1 Exp number, first 60 AAs: 19.54804

# WP_042383306.1 Total prob of N-in: 0.92827

# WP_042383306.1 POSSIBLE N-term signal sequence

WP_042383306.1 TMHMM2.0 inside 1 1

WP_042383306.1 TMHMM2.0 TMhelix 2 21

WP_042383306.1 TMHMM2.0 outside 22 211


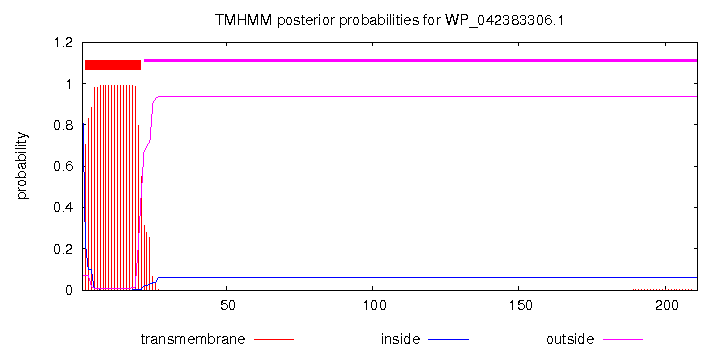


# [plot](http://www.cbs.dtu.dk/services/TMHMM-2.0/tmp/TMHMM_7933/WP_042383306.1.eps) in postscript, [script](http://www.cbs.dtu.dk/services/TMHMM-2.0/tmp/TMHMM_7933/WP_042383306.1.gnuplot) for making the plot in gnuplot, [data](http://www.cbs.dtu.dk/services/TMHMM-2.0/tmp/TMHMM_7933/WP_042383306.1.plp) for plot

gene complement(2835281..2835916)

/locus_tag="CGL_RS13305"

/old_locus_tag="NCgl2577"

CDS complement(2835281..2835916)

/locus_tag="CGL_RS13305"

/old_locus_tag="NCgl2577"

/inference="COORDINATES: similar to AA

sequence:RefSeq:WP_006285906.1"

/note="Derived by automated computational analysis using

gene prediction method: Protein Homology."

/codon_start=1

/transl_table=11

/product="hypothetical protein"

/protein_id="WP_042383306.1"

/translation="MAALLVLLVVIALIIWAVVALRGGSSEPEEEQPNNAVVTSSMES

STTSSSSSKESTTEATTEEETSSAEPTATSTVAADAKKTCELSDLVISASTNQPTFSG

SAQPELFMAVHNPTAVDCEIDLEENKLRFEVYNLATNARIWSDVDCNPAVEDGTSVFP

AGEDRYFQATWSRTTSAPNQCNNRTDVPAGGYYLHTVVGNNPSPAVTFNLT"

25

# WP_003858490.1 Length: 374

# WP_003858490.1 Number of predicted TMHs: 1

# WP_003858490.1 Exp number of AAs in TMHs: 20.20109

# WP_003858490.1 Exp number, first 60 AAs: 20.19552

# WP_003858490.1 Total prob of N-in: 0.93408

# WP_003858490.1 POSSIBLE N-term signal sequence

WP_003858490.1 TMHMM2.0 inside 1 16

WP_003858490.1 TMHMM2.0 TMhelix 17 39

WP_003858490.1 TMHMM2.0 outside 40 374


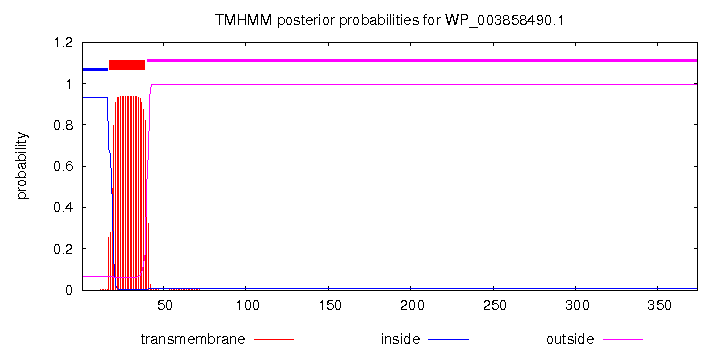


# [plot](http://www.cbs.dtu.dk/services/TMHMM-2.0/tmp/TMHMM_7933/WP_003858490.1.eps) in postscript, [script](http://www.cbs.dtu.dk/services/TMHMM-2.0/tmp/TMHMM_7933/WP_003858490.1.gnuplot) for making the plot in gnuplot, [data](http://www.cbs.dtu.dk/services/TMHMM-2.0/tmp/TMHMM_7933/WP_003858490.1.plp) for plot

gene 963813..964937

/locus_tag="CGL_RS04555"

/old_locus_tag="NCgl0872"

CDS 963813..964937

/locus_tag="CGL_RS04555"

/old_locus_tag="NCgl0872"

/inference="COORDINATES: similar to AA

sequence:RefSeq:WP_003858490.1"

/note="Derived by automated computational analysis using

gene prediction method: Protein Homology."

/codon_start=1

/transl_table=11

/product="resuscitation-promoting factor"

/protein_id="WP_003858490.1"

/translation="MAPHQKSRINRINSTRSVPLRLATGGVLATLLIGGVTAAATKKD

IIVDVNGEQMSLVTMSGTVEGVLAQAGVELGDQDIVSPSLDSSISDEDTVTVRTAKQV

ALVVEGQIQNVTTTAVSVEDLLQEVGGITGADAVDADLSETIPESGLKVSVTKPKIIS

INDGGKVTYVSLAAQNVQEALELRDIELGAQDRINVPLDQQLKNNAAIQIDRVDNTEI

TETVSFDAEPTYVDDPEAPAGDETVVEEGAPGTKEVTRTVTTVNGQEESSTVINEVEI

TAAKPATISRGTKTVAANSVWDQLAQCESGGNWAINTGNGFSGGLQFHPQTWLAYGGG

AFSGDASGASREQQISIAEKVQAAQGWGAWPACTASLGIR"
