# Supplementary material for: Genome-Scale Mining of Novel Anchor Proteins of Corynebacterium glutamicum
Source: Front Microbiol. 2022 Feb 4;12:677702. doi: 10.3389/fmicb.2021.677702 (PMC8854784; doi:10.3389/fmicb.2021.677702)
Supplement: Supplementary File 1 — Tied-mixture hidden Markov models predicted the results for the 25 possible anchor proteins of Corynebacterium glutamicum. [file Data_Sheet_2.docx]

Supplementary Table S1. Reagent used in this study.

| Name | Ingredient |
| --- | --- |
| 10×PBS(Phosphate buffered saline) | 80g L^-1^ NaCl, 2g L^-1^ KCl, 14.2g L^-1^ Na2HPO4, 2.4g L^-1^ KH2PO4, adjust the pH of the solution to 7.4 with HCl |
|  |  |
| CGXII | 40g L^-1^ glucose, 20g L^-1^ammonium sulfate, 5g L^-1^ urine  Vegetarian, 1g L^-1^potassium dihydrogen phosphate, 1g L^-1^ dipotassium hydrogen phosphate, 0.25g L^-1^magnesium sulfate heptahydrate, 10mg L^-1^calcium chloride, 10mg L^-1^iron sulfate heptahydrate, 10mg L^-1^manganese sulfate monohydrate, 1mg L^-1^ zinc sulfate heptahydrate, 0.2mg L^-1^copper sulfate anhydrous, 0.02mg L^-1^ nickel chloride hexahydrate, 30 mg L^-1^protocatechuic acid, 42g L^-1^ MOPs |

Supplementary Table S2. Primers used in this study

| Primer name | Primer sequence |
| --- | --- |
| NCgl0550 F | GAAAGGAGGCCCTTCAGGTGAATATGCGCCGTCGATCC |
| NCgl0550 R | CTTATCGTCATCATCCTTGTAATCTTAAGATGCTGGGTTGTCGATG |
| NCgl0633 F | GCATGGACGAGCTGTACAAGATGCGTTTTTCTCGAGTTCT |
| NCgl0633 R | TCGTCATCATCCTTGTAATCTTACTGGCTGTGCTTGGACT |
| NCgl2115 R | CGTCATCATCCTTGTAATCTTAAGTGATAACACTTGAGCTAGC |
| NCgl2115 F | ATGGACGAGCTGTACAAGATGCGCAGACTCATCGCGGTTA |
| NCgl1460 F | CATGAAAGGAGGCCCTTCAGGTGGAACAGCAAAATAAGCG |
| NCgl1460 R | CTTATCGTCATCATCCTTGTAATCAGCGTTGCTCTGAGTGTTTTCG |
| NCgl1307 F | ATGAAAGGAGGCCCTTCAGTTGAAGGATTACGCGGTGCAT |
| NCgl1307 R | CTTATCGTCATCATCCTTGTAATCGTTCAGTTCTTCACGGCATTG |
| NCgl1876 F | ATGAAAGGAGGCCCTTCAGATGTCTGCAAAGCGTACTTTTAC |
| NCgl1876 R | CTTATCGTCATCATCCTTGTAATCGCTTGCGTCGAGGAAGGAGAG |
| NCgl2562 F | ATGAAAGGAGGCCCTTCAGATGAGCACCACGATTACTCGC |
| NCgl2562 R | CTTATCGTCATCATCCTTGTAATCTTCAGTAGTTGCAACACCTGAG |
| NCgl0067 F | ATGAAAGGAGGCCCTTCAGATGTCTGTTGGTGGATCCGACTG |
| NCgl0067 R | CTTATCGTCATCATCCTTGTAATCCTCCATTACTCCCGGTAGTTTCAC |
| NCgl2291 F | CATGGACGAGCTGTACAAGATGAGCAAGCGTGAAGAATCAAT |
| NCgl2291 R | CTTATCGTCATCATCCTTGTAATCTTAGCTGTGCTGCGTGGTAAGCAG |
| NCgl1147 F | ATGAAAGGAGGCCCTTCAGTTGTTAACAGCTGTTTTGTCTTTAAT |
| NCgl1147 R | CTTATCGTCATCATCCTTGTAATCGCGATTCTCCTTCGGAGCGGGG |
| NCgl1998 F | ATGAAAGGAGGCCCTTCAGATGATTTCCCGACTTCTCCAATTG |
| NCgl1998 R | CTTATCGTCATCATCCTTGTAATCGGGATTTTTGATTTCTTCCAGGTCAATTA |
| NCgl2206 F | ATGAAAGGAGGCCCTTCAGGTGGAATCCTCAGTTATATGGTTG |
| NCgl2206 R | CTTATCGTCATCATCCTTGTAATCACCTTCGTTGTCCTCGCTGGGC |
| NCgl0743 F | ATGAAAGGAGGCCCTTCAGATGGGCCGAATGAAAAACGATGG |
| NCgl0743 R | CTTATCGTCATCATCCTTGTAATCTTTGTCATTTACCTCCTCGCTAAAT |
| NCgl0042 F | ATGAAAGGAGGCCCTTCAGGTGAACCGCTCGATTCGAATCAC |
| NCgl0042 R | CTTATCGTCATCATCCTTGTAATCAAATCCTCCGGCTGCCTGAAGC |
| NCgl0289 F | ATGAAAGGAGGCCCTTCAGATGACAAGCAGTGCAAAGTGGTC |
| NCgl0289 R | CTTATCGTCATCATCCTTGTAATCCGCATTCTGCAGCGCCCCTGCC |
| NCgl1250 F | ATGAAAGGAGGCCCTTCAGATGAAAACAAAGAAGCAGTCCCG |
| NCgl1250 R | CTTATCGTCATCATCCTTGTAATCCTGCGGGAAGGGCTCTAGATCAC |
| NCgl2775 F | ATGAAAGGAGGCCCTTCAGATGAGGAAAACCATCACCGTTAT |
| NCgl2775 R | CTTATCGTCATCATCCTTGTAATCTCCGTTGTCGATGAGGTTGGTC |
| NCgl2610 F | ATGAAAGGAGGCCCTTCAGATGAGCTCCCGAAACTACCGAAG |
| NCgl2610 R | CTTATCGTCATCATCCTTGTAATCTTTCACGCCACCGCCGGAACCG |
| NCgl0136 F | ATGAAAGGAGGCCCTTCAGATGCGTAATCAAACAATCGCTGC |
| NCgl0136 R | CTTATCGTCATCATCCTTGTAATCTAATGTCGCTTTCACTTTAGCAGC |
| NCgl0661 F | ATGAAAGGAGGCCCTTCAGATGAAGAAGGCCATGAGAGCTG |
| NCgl0661 R | CTTATCGTCATCATCCTTGTAATCAAAATAAAACTGAGTTGTCGCCCAC |
| NCgl0717 F | ATGAAAGGAGGCCCTTCAGATGAAAACAGAAACTCGACGAGCCCTC |
| NCgl0717 R | CTTATCGTCATCATCCTTGTAATCGCTCGTGGCAGTTGGTTCCAC |
| NCgl1361 F | ATGAAAGGAGGCCCTTCAGATGGCTAAACGACGTGGAAGAGGC |
| NCgl1361 R | CTTATCGTCATCATCCTTGTAATCCTGCGCTGGCGCTGCTGCAGTT |
| NCgl1682 F | ATGAAAGGAGGCCCTTCAGATGGCGATTAAAGGCGCAATGC |
| NCgl1682 R | CTTATCGTCATCATCCTTGTAATCCTGTATCGCAGCAGCAAGTTCC |
| NCgl2577 F | ATGAAAGGAGGCCCTTCAGATGGCAGCGCTCCTAGTTCTGCT |
| NCgl2577 R | CTTATCGTCATCATCCTTGTAATCAGTTAGGTTAAAGGTCACCGCTGG |
| NCgl0872 F | ATGAAAGGAGGCCCTTCAGATGGCGCCCCATCAGAAGTCACG |
| NCgl0872 R | CTTATCGTCATCATCCTTGTAATCTCGGATGCCCAAGCTTGCGGTG |
| pEC F | GATTACAAGGATGATGACGATAAGGGCTGTTTTGGCG |
| pEC R | CTGAAGGGCCTCCTTTCATGGTCTGTTTCCTGTGTG |
| EGFP F-N site | GATTACAAGGATGATGACGATAAGATGGTGAGCAAGGGCGAGGA |
| EGFP R-N site | TCGTCATCATCCTTGTAATCTTACTTGTACAGCTCGTCCATG |
| EGFP F-C site | ATGAAAGGAGGCCCTTCAGGATTACAAGGATGATGACGA |
| EGFP R-C site | CTTGTACAGCTCGTCCATGCCG |
| mCherry-pEC F | GATTACAAGGATGATGACGATAAGATGGTTTCCAAGGGCGAGGAGGAC |
| mCherry-pEC R | ATGCTTATCGTCATCATCCTTGTAATCTTACTTGTAGAGTTCGTCCATG |
| pEC F-Amy | GGCTGTTTTGGCGGATGAGAGAAGATTTTCAGCCT |
| Amy F | GATTACAAGGATGATGACGATAAGATGTTTGCAAAACGATTCAAAACCTCTTTACTGCC |
| Amy R | TCTTCTCTCATCCGCCAAAACAGCCTCAATGGGGAAGAGAACCGCTT |
| pEC-JD F | CACTGCATAATTCGTGTCGCTCAAGGCGCACTCC |
| pEC-JD R | TGTCCTACTCAGGAGAGCGTTCACCGAC |
| EGFP-JD R-N site | GTCGCCGTCCAGCTCGACCAGGATG |
| EGFP-JD F-C site | GATCACTCTCGGCATGGACGAGCTG |
| Amy JD R | GAACGACCAATTCCATGCATGAAGAATGGTTCCGC |
| mCherry JD R | CTTGTAGGTGGTCTTAACCTCAGCGTCGTAGTGACCG |

Note: The primers used in this study were synthesized by Guangzhou Kinco Biological Company. Snapgene was used to design primers, analyze and draw plasmid maps.

Supplementary Table S3. The prediction results of the known endogenous anchor proteins of *C. glutamicum* made by TMHMM

| Protein_id/ GeneID | Product | TMHMM parameter |
| --- | --- | --- |
| WP_003858702.1/ NCgl1337 | SGNH/GDSL hydrolase family protein | Length: 324  Number of predicted TMHs: 1  Exp number of AAs in TMHs: 21.10965  Exp number, first 60 AAs: 21.10464  Total prob of N-in: 0.95159  POSSIBLE N-term signal sequence  inside 1 6  TMhelix 7 29  outside 30 324 |
| WP_011014245.1/ NCgl1221 | mechanosensitive ion channel | Length: 533  Number of predicted TMHs: 3  Exp number of AAs in TMHs: 67.2569  Exp number, first 60 AAs: 22.6999  Total prob of N-in: 0.65627  POSSIBLE N-term signal sequence  inside 1 12  TMhelix 13 35  outside 36 63  TMhelix 64 86  inside 87 92  TMhelix 93 115  outside 116 533 |
| WP_003856752.1 | PorB | Length: 126  Number of predicted TMHs: 0  Exp number of AAs in TMHs: 14.82472  Exp number, first 60 AAs: 14.78601  Total prob of N-in: 0.74802  POSSIBLE N-term signal sequence  outside 1 126 |
| WP_003856749.1 | PorC | Length: 123  Number of predicted TMHs: 0  Exp number of AAs in TMHs: 1.31056  Exp number, first 60 AAs: 1.30244  Total prob of N-in: 0.35847  outside 1 123 |
| WP_011265995.1 | PorH | Length: 57  Number of predicted TMHs: 0  Exp number of AAs in TMHs: 0.35918  Exp number, first 60 AAs: 0.35918  Total prob of N-in: 0.10069  outside 1 57 |


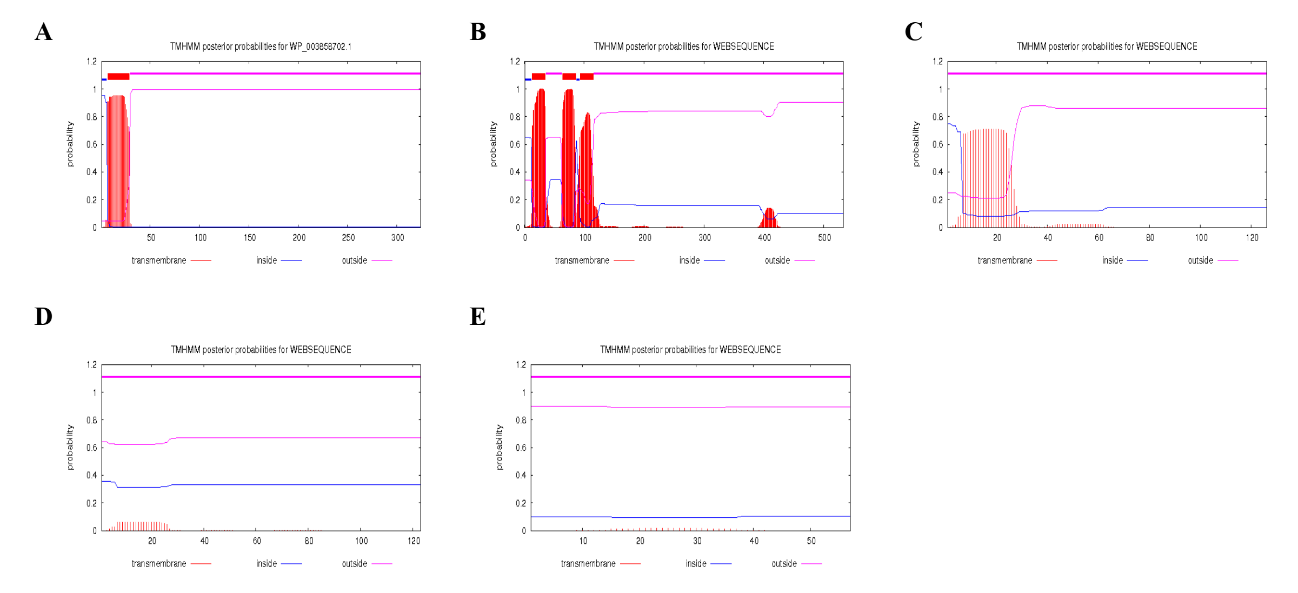


Supplementary Figure 1


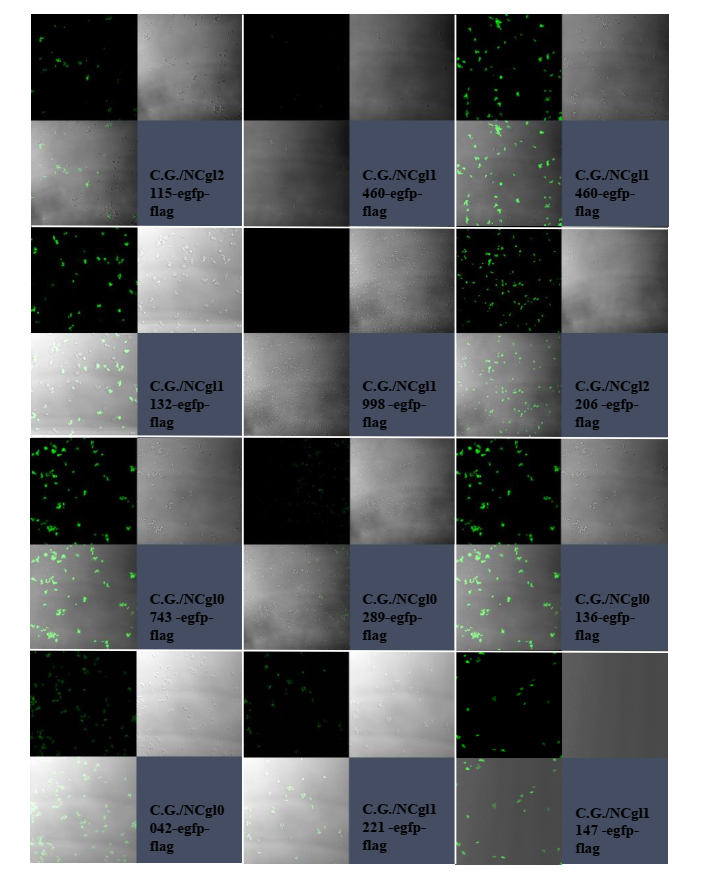


Supplementary Figure 2


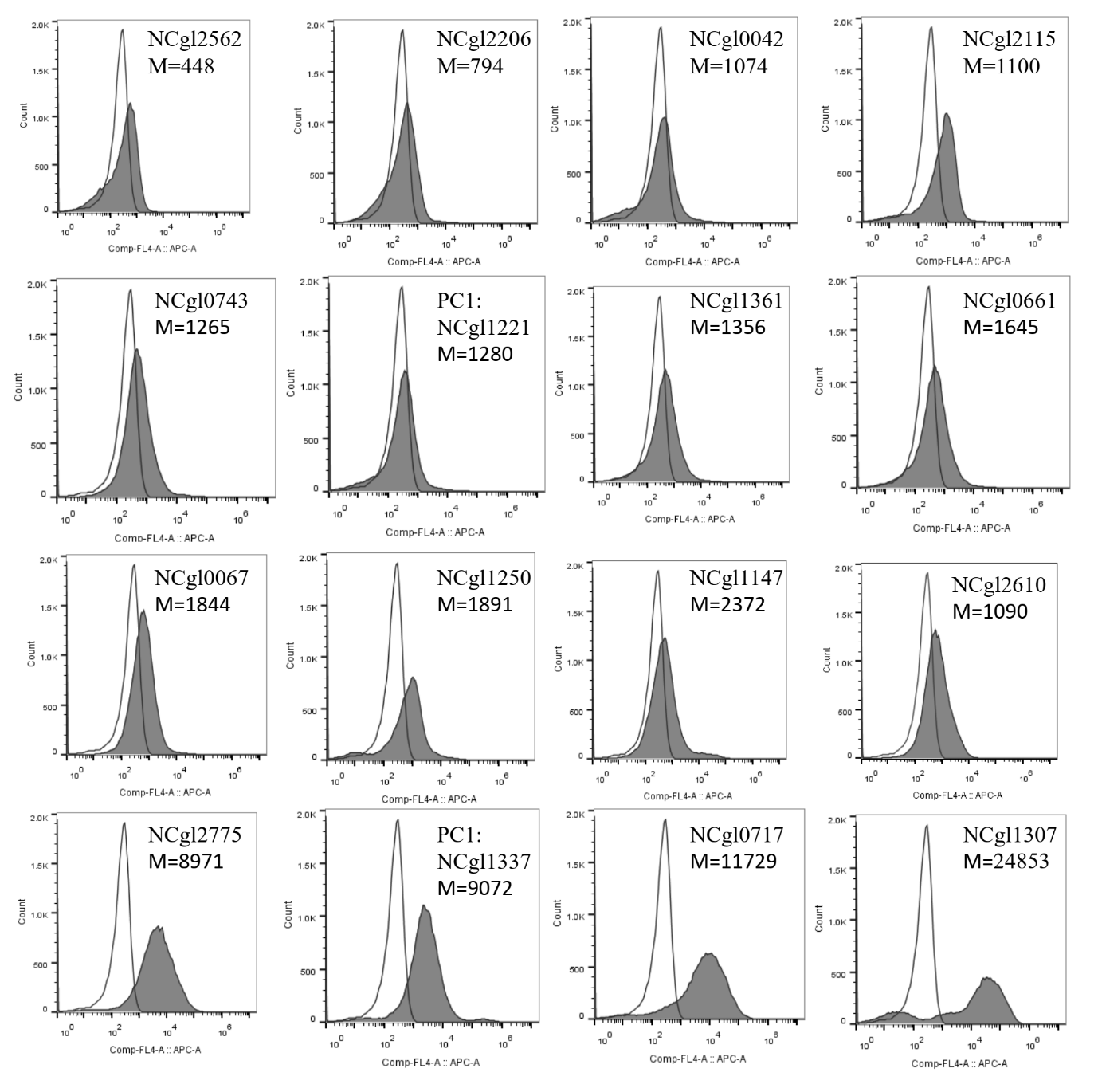


Supplementary Figure 3


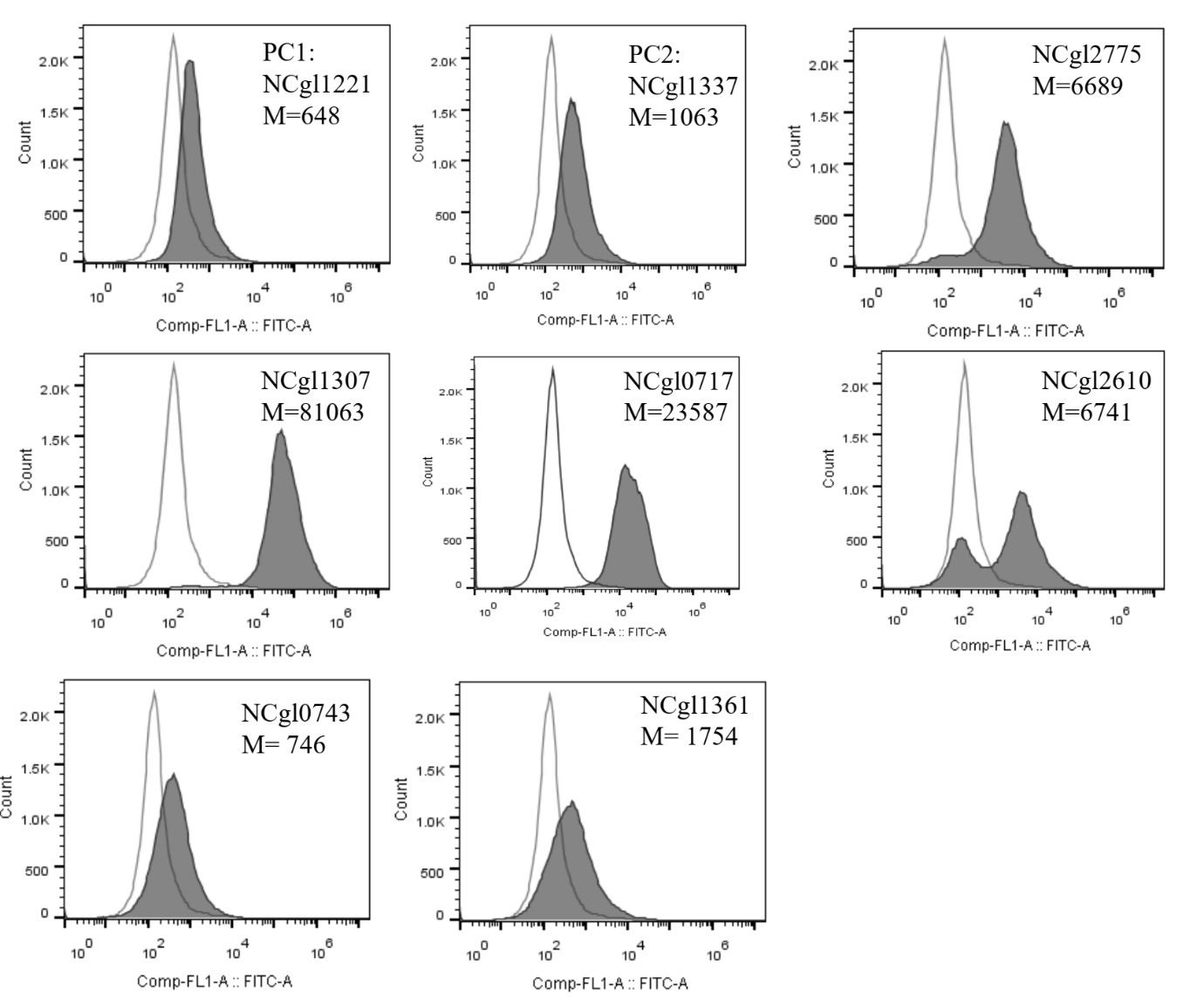


Supplementary Figure 4


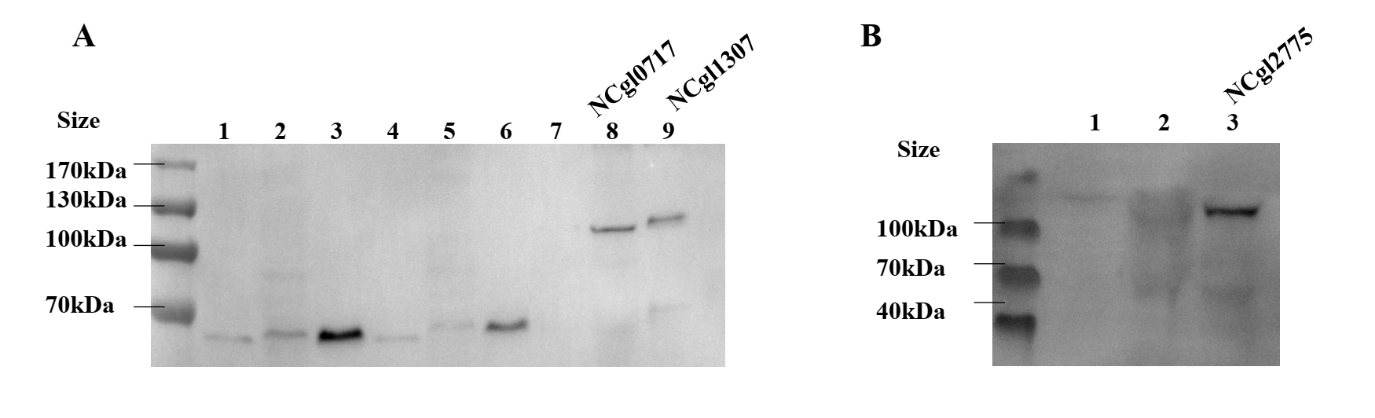


Supplementary Figure 5
